# Robot-Assisted Study of a Near-Infrared Dye in Perovskite Solar Cells

**DOI:** 10.1021/acsami.6c05093

**Published:** 2026-06-30

**Authors:** Bingyu Lei, Per H. Svensson, Lars Kloo

**Affiliations:** Applied Physical Chemistry, Department of Chemistry, 7655 KTH Royal Institute of Technology, Stockholm SE-114 28, Sweden

**Keywords:** automated experimentation, robot-assisted fabrication, perovskite solar cells, near-infrared dye, density-functional theory

## Abstract

The investigation of dyes in perovskite solar cells (PSCs) is an emerging field, since dyes may introduce a range of effects beyond conventional light harvesting. However, the large number of possible materials and processing combinations make systematic investigation challenging. This study presents a hybrid and transferable strategy combining automated workflows and supporting manual evaluation to gain insights into the effects of dyes in PSCs. Using a near-infrared (NIR) dye and a typical lead halide perovskite, an automated synthesis-characterization workflow is demonstrated to be suitable for the evaluation of the effects of a dye either as a precursor additive or as a post-treatment material using photoluminescence as the key performance parameter. In addition, the automated synthesis-device fabrication-evaluation workflow can provide guidance for identifying a suitable concentration range of dye additives in precursor solutions. Computational results at density-functional theory (DFT) level and manually performed experiments suggest that the dye MK245 interacts with the perovskite and modifies its local or interfacial electronic environment. Integration of MK245 can significantly improve the device stability both in mesoscopic triple-layer perovskite solar cells and in conventional thin-film solar cells.

## Graphical abstract

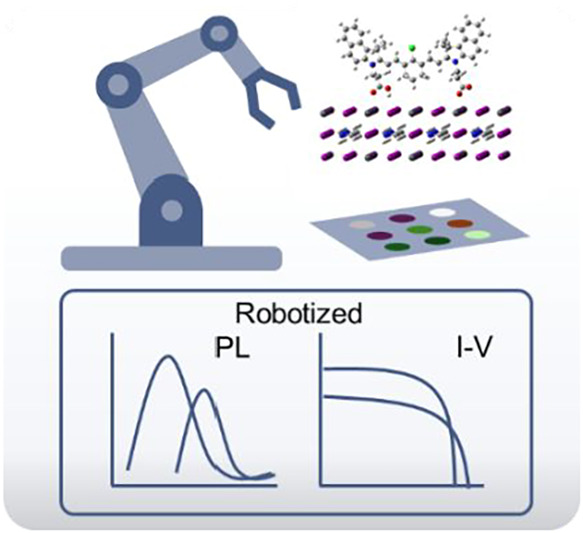

## 1 Introduction

Since the first investigation of organometal halide perovskites as light absorbers in a dye-sensitized solar cell (DSSC) architecture, metal-halide perovskite solar cells (PSCs) have attracted tremendous research attention with rapid progress in power conversion efficiencies (PCEs) from 3.8%^1^ to the present record of >27%.^2^ This is attributed to excellent photoelectric properties of the perovskite materials and significant efforts from researchers on the composition design, interface optimization, charge-transport materials development and device engineering.^3–6^ In addition, ongoing studies also target the device stability issues, exploring new alternatives to Pb-based perovskite materials and improving the commercialization potential of PSCs.^7–11^

Beyond the well-known development history, dyes have also played a significant role in inspiring and advancing the development of perovskite solar cells and related areas in different ways. For example, a spectral splitting photovoltaic device based on a perovskite solar cell and a panchromatic dye-sensitized solar cell has been reported to exhibit a broad response into the near-infrared (NIR) region and displayed a conversion efficiency of 21.5%.^12^ In addition, dyes have been incorporated into PSCs to serve different functions including light-absorption extension, surface passivation, interface engineering, stability enhancement and combined multifunctions.^13–20^ Our group has also reported perovskite-like materials with internal dye-sensitization, expanding the compositional and structural space of new perovskite-like materials.^21^

Despite the promising performance and interesting observations, a large number of dyes remain unexplored, and there is still a lack of comprehensive discussion on their performance in PSCs. The vast diversity of dyes and perovskite materials that can be selected makes this evaluation a time-consuming and highly repetitive procedure, which highlights the demand for a less time- and labor-intensive strategy that can screen and identify interesting candidates for further detailed study. In many materials research areas with similar challenges, one of the solutions that has been explored is automated screening using robotic workflows. Robotization has shown the capability to assist the exploration and optimization of pharmaceuticals, organic chemicals, inorganic solids, semiconducting polymers, photovoltaic materials and so on.^22–27^ The standardized evaluation workflow also opens the possibility for establishing a materials gene database, allowing systematic analyses and inverse design of new materials.^28–31^

Herein, we have developed an automated synthesis-characterization workflow based on our previously reported AURORA robotic system for the study of the effects of incorporating a dye into PSCs.^32^ In contrast to our previous work that explored internal dye incorporation within the perovskite crystal lattice to enable intrinsic sensitization and new hybrid phases, this work focuses on the functionalization strategies that preserve the underlying 3D perovskite structure and enable compatibility with existing device architectures.^21^ This also allows a broader selection of dye molecules in terms of charge or spin state and molecular size, since it does not require strict compliance with the ionic radii and symmetry requirements of the expected perovskite crystal lattice. The approach also incorporates a previously reported automated device evaluation workflow and complementary manual characterization. We demonstrate the applicability of such a hybrid workflow using a near-infrared dye, MK245 (hereafter referred to as MK), and methylammonium lead triiodide (MAPI) as the model materials with the more general aim to show the capability of an automated approach to be generalized to a wide range of solar cell materials. The results also suggest that the example dye can improve the stability of perovskite solar cells both in mesoscopic triple-layer scaffolds and in conventional thin-film n-i-p device configurations.

## 2 Results and Discussion

### 2.1 Automated Synthesis-Characterization Workflow

Based on AURORA,^32^ our recently reported automatic robotic platform for materials discovery, an automated synthesis-characterization workflow was designed to combine robotic liquid handling, film deposition and rapid throughput photoluminescence (PL) measurements. PL characterization has proved to be a powerful tool to investigate film quality, phase growth behavior and stability of perovskite materials, and it has been introduced to many robotic platforms by integrating a well-plate reader with a PL measurement function.^33–35^ In this work, ZrO_2_ film arrays were introduced as the substrates for film deposition and PL measurement. As a commonly used insulating spacer scaffold material in mesoscopic perovskite solar cells, ZrO_2_ provides a stable mesoporous framework for perovskite crystallization, and separates the electron- and hole-transport layers due to its wide band gap. Applying ZrO_2_ substrates in the materials characterization workflow can replicate the crystallization process in mesoscopic devices or on other mesoporous charge-transport layers. The intrinsic hydrophilicity also ensures efficient solution infiltration without additional surface treatment. In order to fit the well-plate reader, ZrO_2_ films were printed on glass substrates with a pattern corresponding to a 24-well plate (Figure S1), enabling preparation and characterization of up to 20 films within a single batch. A liquid-handling robot integrated with a hot plate was used for solution preparation and film casting. The ZrO_2_ array plate was transferred between the synthesis module and the characterization module by a robot arm equipped with a vacuum gripper. The included modules are shown in Figure S2 and are illustrated in Figure 1a. The workflow was controlled by an orchestrator Python script and can operate in a fully automated mode, enabling standardized and unattended comparative studies under consistent experimental conditions. Although not implemented in this work, efforts are underway to increase the throughput by adopting 48-well-plate-like and 96-well-plate-like configurations. In addition, owing to its modular design, the workflow is readily iterative and adaptable, allowing further integration of real-time data analysis and design of experiment (DoE) algorithms, such as Bayesian optimization.^35–37^ It is also worth emphasizing that, although a dye is used as the model system in this work, the workflow is equally applicable to other functional molecules, such as organic cations of different sizes, which will be further explored in future studies.

**Figure 1:**
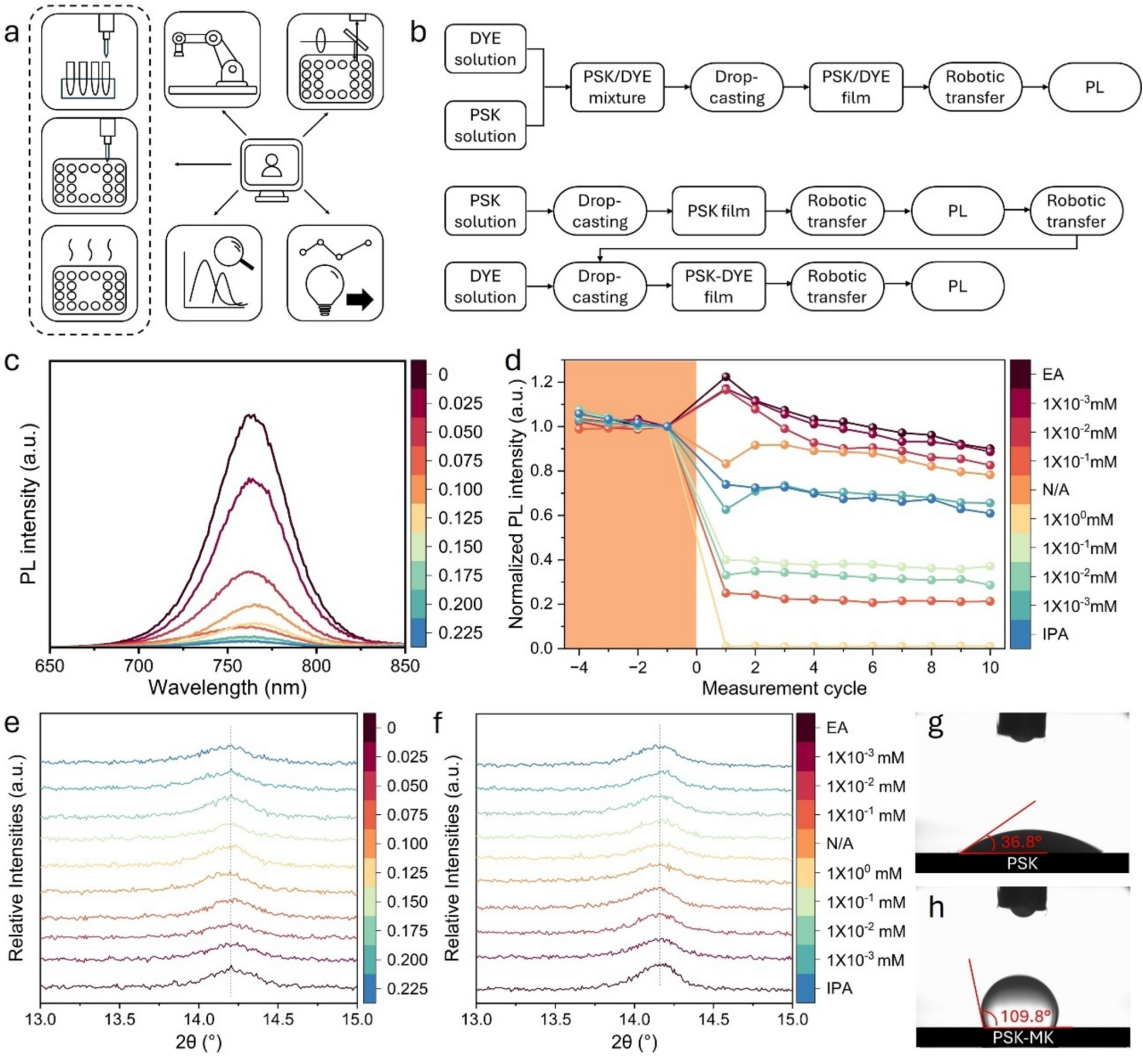
(a) Schematic diagram and (b) flowcharts of the automated workflow used in this work. (c) PL and (e) PXRD result from the AW. (d) PL and (f) PXRD result from the PTW. (g, h) Water contact angles of pristine perovskite (PSK) and MK-treated perovskite (PSK-MK) films.

Two workflows were designed to study the impact of the dye on the perovskite films and are referred to as the “additive workflow” (AW) and the “post-treatment workflow” (PTW), respectively, in the following discussion (Figure 1b). First, to study the dye as an additive in perovskite precursor solutions, dye solutions with different concentrations were prepared and mixed with the perovskite precursor stock solutions (Tables S1 and S2). Certain volumes of the resulting mixtures were then dropped onto different ZrO_2_ substrates and heated for a set time to generate circular films on the well-plate-like glass substrate. Next, the robot arm transferred the ZrO_2_ array plate to a metal cooling stage and then to the plate reader for PL measurements. After the PL measurements, the robot arm transferred the ZrO_2_ plate back to the liquid-handling robot, while the PL data were saved. In addition, the dye was also studied as a post-treatment material by expanding the first workflow into two cycles. In the first cycle, pure perovskite solutions were drop-casted onto the ZrO_2_ scaffolds to prepare intrinsic perovskite films. During the second cycle, different post-treatment solutions were prepared (Table S3) and subsequently dispensed onto the ZrO_2_-perovskite films. PL data before and after post-treatment were collected, and the relative PL intensity changes from the post-treatment were extracted.

From the results in Figure 1c, it can be noted that the perovskite samples incorporating the example dye, MK, exhibit significantly reduced PL intensity as compared to the pristine perovskite sample. Compared with other investigated dyes, including Victorian Blue B (VBB), Quinaldine Red (QR) and Indigo, MK shows the most pronounced PL quenching behavior under the investigated conditions (Figure S3). It is worth noting that both VBB and QR have been investigated in our previous work as implicit dye sensitizers to construct perovskite-like structures together with PbI_2_.^21,38^ Both dyes exhibit enhanced PL intensities at low concentrations, suggesting possible passivation-like effects and potentially different dye-perovskite interaction mechanisms. However, further investigation is required for confirmation and will be presented in a future work. From the results in Figure 1d, it can be observed that a perovskite sample coated by MK (1 mM in isopropanol, IPA) also shows a dramatic PL reduction. After dilution by different solvents, the effect becomes gradually like that of pure solvent treatment. A reduction in PL intensity is particularly sensitive to multiple competing processes in perovskite thin films, since steady-state PL reflects the balance between radiative recombination, trap-assisted nonradiative recombination, and possible interfacial or local carrier redistribution. Consequently, PL quenching cannot be directly attributed to a single mechanism without further analyses. Since MK induced a consistent and concentration-dependent PL reduction under both additive and post-treatment conditions, this motivated additional structural and electronic characterization to better understand the origins of this behavior.

### 2.2 Additional Characterization

In order to further investigate the interaction between the MK dye and the perovskite films, we first performed complementary measurements directly on the robot-fabricated films. Figures S4 and S5 show the powder X-ray diffraction (PXRD) traces of the robot-synthesized samples. All samples exhibit highly similar diffraction peaks at 2θ values larger than 15°. These peaks correspond to ZrO_2_ and FTO (Figure S6) from the substrate, which indicate that the use of less PXRD-active substrate materials represents a future potential improvement of characterization. The peak located at 2θ = 14.2° is nevertheless characteristic, corresponding to the (110) crystal plane of the tetragonal phase of MAPI. For all the samples originating from both the AW and the PTW, this peak does not show any observable shift (Figure 1e,f) nor any systematic peak broadening (Tables S4 and S5), indicating that the incorporation of the dye does not induce detectable changes in the crystal structure of MAPI.

For samples obtained from the AW, there is no additional PXRD peak observed except that from the background or the perovskite material itself, suggesting that no new crystalline products are formed as a result of the incorporation of MK into the precursor solutions within the concentration range investigated. While for samples from the PTW, as shown in Figure S5, a slightly more intense peak located at 2θ = 12.7° can be observed for samples treated by solutions in IPA as compared to those treated by solutions diluted with ethyl acetate (EA), most likely originating from precipitated PbI_2_ because of the high solubility of methylammonium iodide (MAI) in IPA. This may also explain the generally lower PL intensity recorded from samples that were treated by IPA-based solutions. In some cases, this effect can promote a perovskite surface modification or reconstruction, thus improving the film quality or the anchoring process of the materials used in the post-treatment.^39,40^ However, to achieve optimal results, post-treatment conditions need to be further adjusted for different materials, and an automated workflow will offer advantages in this regard. Some additional diffraction peaks can be observed for samples treated by EA or very low concentrations of MK in IPA, and these can be attributed to the formation of a solvated complex with *N*,*N*-dimethylformamide (DMF). This is likely due to the insufficient removal of DMF during the first cycle of synthesis. Apart from these new diffraction peaks, no new products as a consequence of MK could be identified using PXRD after the post-treatment.

In addition, the water contact angle measurements indicate a more hydrophobic surface after dye coating, which may contribute to improved resistance against water-induced degradation of the perovskite film (Figures 1g,h).^41^ Moreover, UV−vis diffuse reflectance spectra (DRS) of the robot-fabricated films reveal an extended absorption range into the near-infrared region after dye incorporation (Figure S7), which is an expected outcome of incorporating a NIR dye into the system.

Additional characterization was performed on the dye MK. As shown in Figure S8, MK belongs to the cyanine family of dyes with a very similar structure as the dye NK-6037.^42,43^ Both dyes have been studied for NIR-DSSCs. The molecular structure was further confirmed by single-crystal X-ray diffraction (Figures 2a and S9 and Table S6). The corresponding PXRD pattern can be simulated based on the single-crystal structure and is shown in Figure S10. In the crystal structure, the dye is observed in its protonated hydrochloride form containing two carboxylic acid groups and a chloride counterion. The incorporation of the chloride anion is most likely an unwanted consequence of the use of hydrogen chloride during the synthesis of the dye. Nevertheless, according to relevant literature and considering the application conditions, such as a system containing the Lewis acid Pb^2+^, our further analysis and discussion is based on the neutral structure, in which one of the carboxylic acid groups is deprotonated.^42^ UV−vis absorption and PL spectra of the dye in an IPA solution (Figure 2b) suggest an optical band gap of around 1.45 eV and a typically small Stokes shift of 32 nm. This aligns well with the results from the density-functional theory (DFT) computations (Figure 2c), from which the highest occupied molecular orbital (HOMO) and lowest unoccupied molecular orbital (LUMO) are calculated to be −4.68 and −3.25 eV at the O3LYP level of theory. Comparative studies using different density functionals were also conducted, and the results are summarized in Table S7. In general, different functionals predict similar molecular properties with minor variations in the computed energy levels. In addition, the inclusion of implicit solvent effects generally shifts the transition to longer wavelengths and the predominant singlet-to-singlet (*S*_0_-*S*_1_) transition emerges as a more pure HOMO-to-LUMO excitation. The electrostatic potential (ESP) map shows that the negative charges tend to accumulate on the −COO^−^ and −COOH groups, suggesting that the MK dye can interact with coordinatively unsaturated Pb^2+^ sites and thereby potentially reduce trap-related recombination.

**Figure 2:**
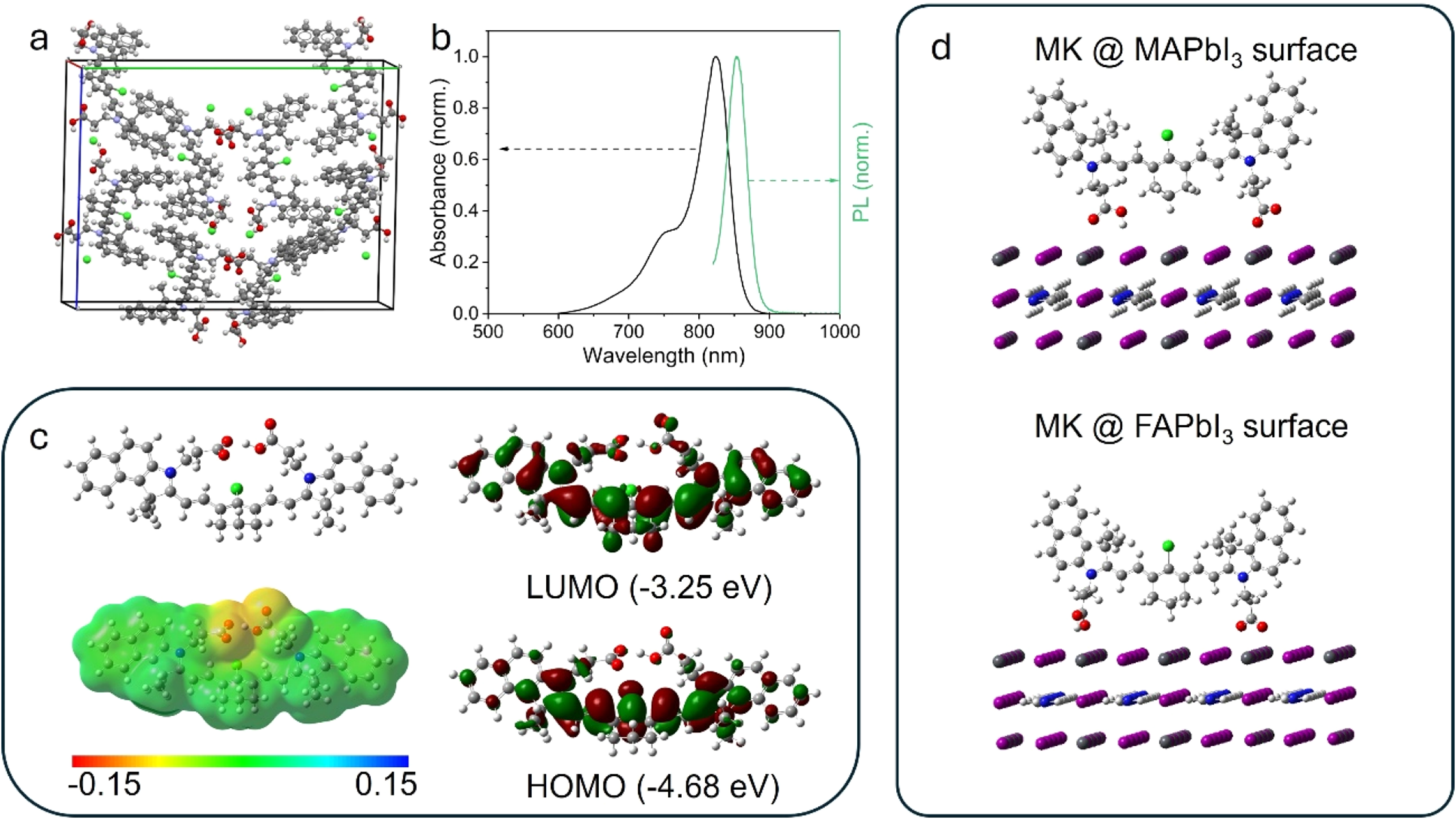
(a) Single-crystal structure of MK as its chloride salt (CCDC 2481131). The compound crystallizes in the orthorhombic crystal system (space group *Pca*2_1_) with unit cell parameters *a* = 7.9900(16) Å, *b* = 35.116(7) Å, *c* = 26.375(5) Å, α = β = γ = 90°. (b) UV−vis absorption spectrum and PL spectrum of MK in an IPA solution. (c) Optimized configuration, molecular orbitals for HOMO and LUMO, electrostatic surface potential distribution of the neutral molecule MK. (d) Structures of the geometrically optimized, neutral MK coordination/adsorption mode on MAPI and FAPI surfaces.

The interface between the perovskite surface and the dye has also been studied using DFT computations for idealized surfaces. In addition to MAPI, formamidinium lead triiodide (FAPI) was also investigated to assess the generality of the dye-perovskite interaction beyond the experimentally used MAPI model system. Slab models consisting of 44 Pb^2+^, 105 I^−^ and 16 monovalent organic cations, methylammonium (MA^+^) and formamidinium (FA^+^) cations respectively, were constructed with the organic cations in the ideal A position of a cubic perovskite structure model (APbI_3_, A = MA or FA, corresponding to the MAPI and FAPI compounds, respectively) rendering an overall slab charge of −1. Images of the two models with MK (monoprotonated, neutral) adsorbed are shown in Figure 2d. During the geometric optimizations, the MAPI/FAPI slab was kept frozen and the atoms in the MK molecule were left fully free. The optimized coordination modes are quite similar in the two systems, although it should be noted that the potential energy surface of adsorption is quite flat offering many coordination possibilities representing local energy minima. It is notable that a starting configuration with MK lying flat on its side on the surface changes into a standing configuration with a lower total energy. The −COOH group preferably coordinates to I^−^ on the top surface (shortest distance of 2.57 Å and 2.44 Å, respectively for the two perovskite materials), whereas the −COO^−^ group displays a quasi-bidentate coordination to Pb^2+^ (shortest distance of 2.56 Å and 2.37 Å, respectively for the two perovskite materials). The adsorption of MK to the perovskite slabs surfaces can thus be attributed to a combination of hydrogen bonding with I^−^ and predominantly electrostatic attraction to the Lewis acid Pb^2+^. These interactions are most likely stronger than the dispersion interaction of an MK lying flat on its side rendering a standing adsorption configuration also in the absence of other close-by dye molecules.

The surface interactions not only enhance the binding stability but also influence the electronic band structure. The HOMO of the MAPI slab is −3.279 eV and of the FAPI model is −3.091 eV. Coordination with MK shifts the HOMO energy to −3.222 eV (shift +0.059 eV) for MAPI and −3.002 eV (shift +0.089 eV) for FAPI. This is also consistent with the experimental observations *vide infra*, which show a similar valence band maximum (VBM) shift upon dye functionalization.

In order to further support the results from robotized workflows and the DFT computations, MAPI films were prepared by spin-coating with and without MK coating. X-ray photoelectron spectroscopy (XPS) was employed to study their interaction at the surface. Figure 3a presents the C 1s spectra for both films. Compared with the spectral features of intrinsic perovskite films, the samples with MK show a higher ratio of the C−C/C–H peak (284.8 eV) and the appearance of a C=O peak (289.1 eV) which can be attributed to the MK dye at the surface. From Figure 3a–c, it can be observed that there is a shift toward lower binding energy for the C−N peak, and both the Pb 4f peaks and I 3d peaks. This suggests that the interaction between the dye and the perovskite surface most likely involves bonding between −COO^−^ from the dye and coordinatively unsaturated Pb^2+^ in the perovskite film, resulting in the observed changes in the chemical environment. In addition, the uncoated MAPI films show Pb^0^ peaks that are absent in the dye-coated samples, indicating that the MK dye can effectively suppress the formation of elemental Pb due to degradation and thereby improve the stability of the perovskite film.^44^ This is consistent with the aforementioned contact-angle results (Figure 1g,h) and the differences in PXRD patterns for fresh and aged films (Figure 3f). More specifically, the dye coating appears to suppress moisture-induced degradation of the perovskite film and the concomitant formation of PbI_2_ and associated Pb^0^ defects, although scanning electron microscopy (SEM) images do not reveal any significant differences in the film morphologies (Figure 3g,h).

**Figure 3:**
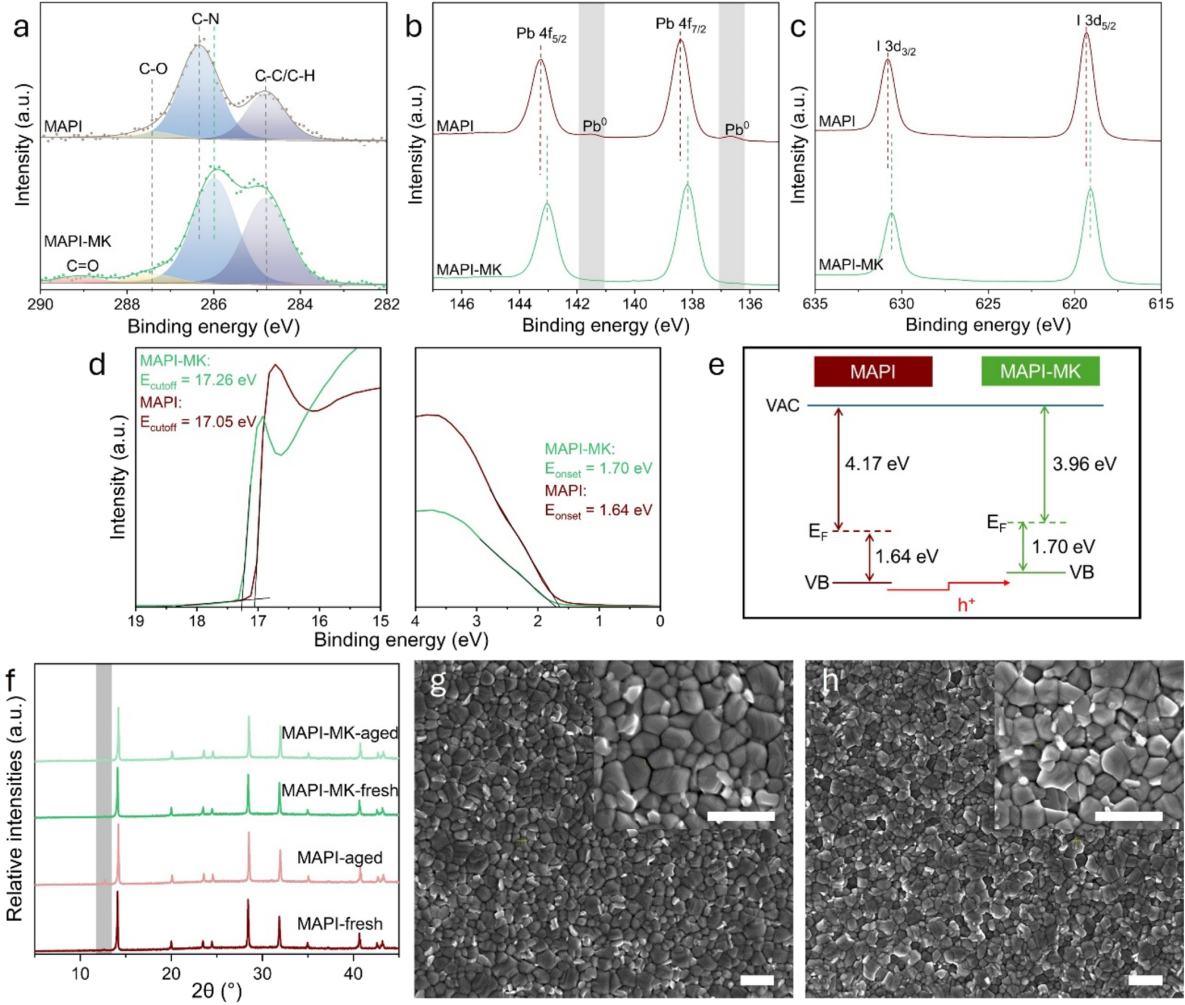
XPS profiles of MAPI with and without MK dye coating, (a) C 1s; (b) Pb 4f; (c) I 3d. (d) He I UPS spectra of MAPI and MAPI-MK. (e) Energy diagram determined from UPS measurements. (f) PXRD traces of fresh and aged MAPI films with or without MK dye coating. SEM images of aged (g) MAPI and (h) MAPI-MK films. The white scale bar corresponds to 1 μm.

Ultraviolet photoelectron spectroscopy (UPS) was used to investigate the impact on the energy levels by dye adsorption. As shown in Figure 3d, the capping of the MK dye increases the work function (WF = *h* *υ* − *E*_cutoff_) from −4.17 eV to −3.96 eV and the distance between the VBM and the Fermi level (*E*_F_) from 1.64 to 1.70 eV, as is estimated from the *E*_onset_. This leads to an overall upward shift of the VBM of the MK-incorporated surface as compared to the pristine MAPI surface, indicating modified interfacial energetics that can facilitate hole transfer or carrier redistribution at the MK-perovskite interface (Figure 3e). Although coordination of MK with coordinatively unsaturated Pb^2+^ may reduce trap-assisted recombination, the resulting electronic structure modification can also promote carrier redistribution, contributing to the observed PL quenching.

### 2.3 Device Evaluation

Based on the robotic workflows and the additional characterization discussed above, the influence of MK incorporation on the photovoltaic performance was further investigated in perovskite solar cells. MAPI was used as the model perovskite material to maintain consistency with previous studies, although it generally exhibits lower conversion efficiencies than more advanced, multicomponent perovskite systems. The incorporation of the MK dye into perovskite solar cells was first studied in our customized robotic solar cell fabrication-evaluation module, adopting an automated workflow including solution preparation, drop-casting onto printed mesoscopic solar-cell arrays with iterated heating, cooling and current density−voltage (*J*–*V*) measurement under 1000 lx of LED-based irradiation.^32^ The workflow is illustrated in Figure 4a. The dye was mixed with perovskite precursor solutions to generate different concentrations from 0 to 7 mM, while maintaining a constant final MAPI concentration of approximately 1.43 M. In two batches of cells, solutions were dispensed at different array coordinates on the solar cell array substrate to minimize the effects of the specific printing coordinates and to enhance the data reliability. The resulting devices were labeled by the concentration of the dye for direct comparison. Details are given in Table S8.

**Figure 4:**
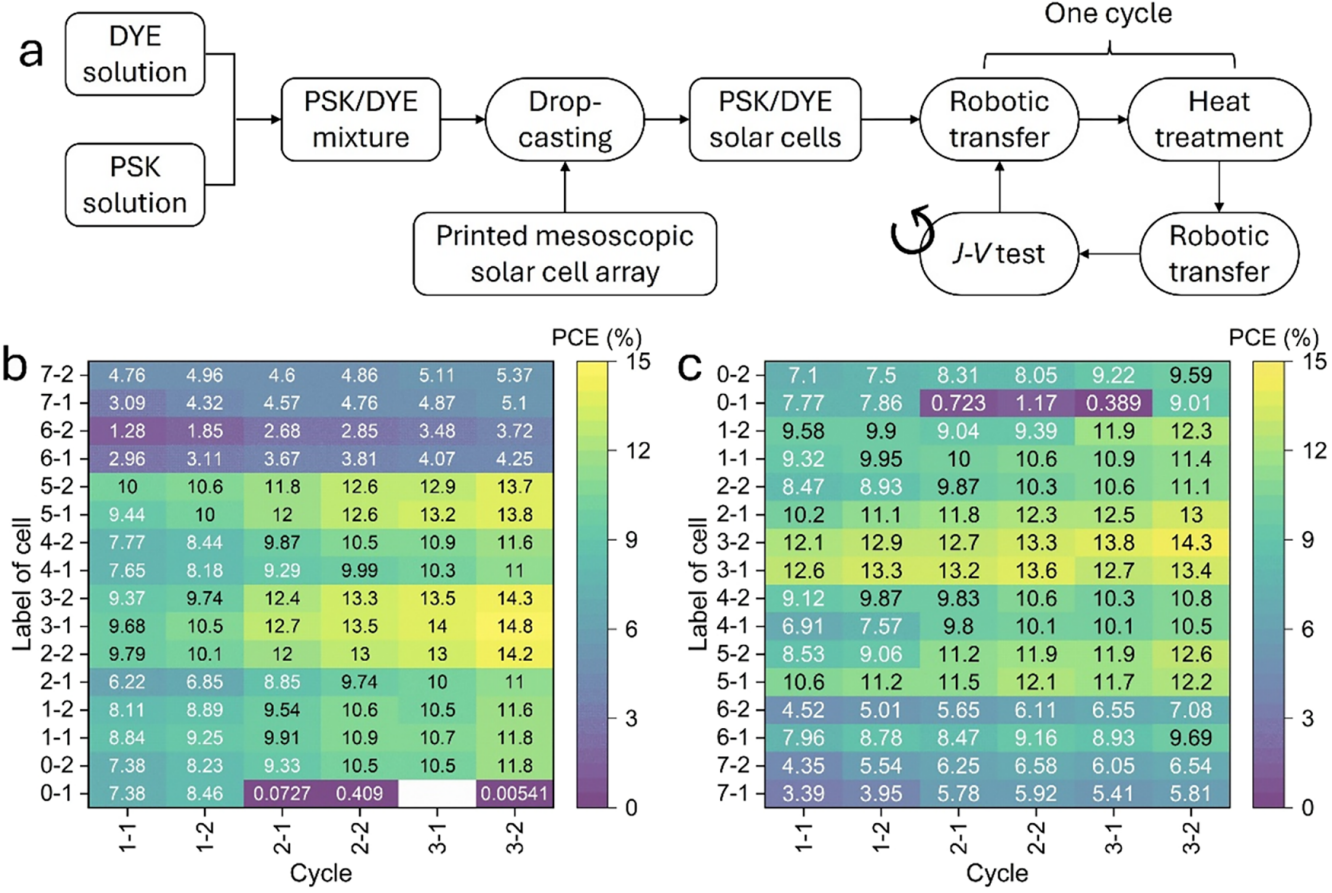
(a) Flowchart of the robotic device fabrication-evaluation workflow. (b, c) extracted PCE from forward scans of the *J*−*V* measurements of two batches of devices. On the abscissa, labels in the format *a*-*b* indicate that the data were collected from the *b* ^th^ *J−V* measurement of the *a* ^th^ cycle; for the labels in the format *i*-*j* on the ordinate, *i* represents the concentration of the solution used and *j* denotes the cell number for each concentration.

Each batch of cells was investigated in three cycles. In each cycle, the substrate plate was heated at 90 °C for 5 min, followed by automatic cooling to 30 °C, additional cooling on a metal block and two rounds of *J*–*V* characterization in the solar cell module. The original current−voltage (*I*–*V)* curves are shown in Figures S11 and S12. Due to the irregularities observed in the reverse scans, which may lead to an overestimation of device performance metrics, particularly the fill factor, PCE values for the robotic workflow were extracted from the forward scans, consistent with our previous study.^32^ The corresponding results for the two batches of cells are shown in Figure 4b,c. Both show that 3 mM of dye added into the precursor solution increases the PCE of the mesoscopic perovskite solar cell under 1000 lx illumination. However, when the concentration is increased to 6 mM, there is a significant reduction of the PCE. This behavior is consistent with the observed concentration-dependent PL quenching results, suggesting that excessive MK incorporation may adversely affect carrier transport and recombination dynamics at elevated concentrations. This also highlights the capability of the automated workflow to identify the effective concentration ranges for precursor additives, which can substantially reduce human labor; particularly when screening large libraries of new materials.

The champion concentrations were also investigated in manually fabricated mesoscopic solar cells using commercial mesoscopic electrodes, which share the same morphology of the inorganic scaffold as the substrates used in the robotic workflow (Figure 5b). The fabricated solar cells were investigated under AM 1.5G 1-sun (100 mW cm^−2^) illumination. The *J*–*V* metrics over time are compared in Figure 5a. The improvement in PCE for fresh cells by dye incorporation is smaller than that observed in the robotic workflow. This is likely due to the much larger active area of the commercial mesoscopic substrate (1.5 cm^2^), which requires stricter control of the crystallization process. As a result, the corresponding devices are more susceptible to variations introduced during manual fabrication, which may partially mask the beneficial effects of the additives. However, the dye-incorporated devices exhibit clearly better long-term stability. After being stored under ambient conditions (20 °C, 40−60% RH) without encapsulation for 20 days, the dye-incorporated devices retained 99% of their initial average fresh PCE, while the control devices dropped to 67% of their initial average PCE. This can be attributed to the barrier effect of the dye, which protects the perovskite from moisture and air-induced degradation.

**Figure 5:**
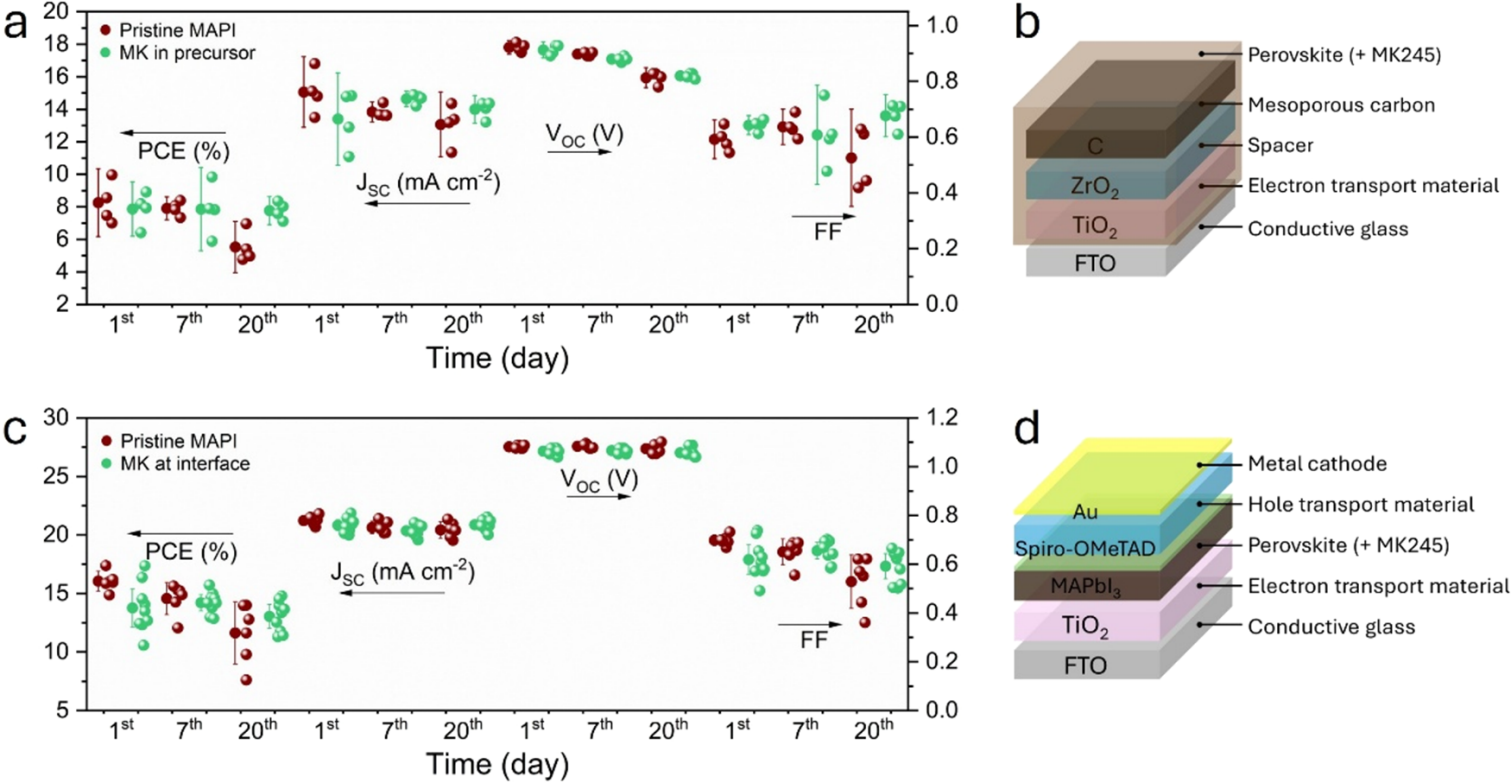
(a) *J*−*V* metrics over time of the manually fabricated mesoscopic solar cells. (Red: MAPI; Green: with 3 mM MK in the precursor solution). (b) Illustrative diagram of the mesoscopic solar cell configuration used in the robotic workflow and the manual device fabrication. (c) *J*−*V* metrics over time of the manually fabricated thin-film solar cells with (green) or without (red) the MK dye at the perovskite/hole transport layer interface. (d) Illustrative diagram of the conventional type of n-i-p solar cells. Devices were stored together under ambient conditions (20 °C, 40−60% RH) without encapsulation and were characterized at the indicated time points.

In order to further explore other possible functions of the dye, Incident-Photon-to-Current Efficiency (IPCE) was recorded (Figure S13). However, the dye-incorporated devices do not show any observable improved IPCE response at the wavelengths where it is expected to introduce enhanced absorption (NIR). This indicates that photons absorbed by the dye do not contribute effectively to the generation of the device photocurrent. The free charge-carrier generation of the dye may be limited, as indicated by the small Stokes shift. This is also likely caused by unfavorable energy-level matching between the dye LUMO and the perovskite conduction bands, most likely preventing efficient charge injection; a general challenge similar to that observed in dye-sensitized solar cells. This indicates that the primary role of the MK dye in the present system is not sensitization but instead defect passivation, electronic structure modulation and device stability enhancement.

Conventional types of PSCs were also fabricated with the configuration of FTO/c-TiO_2_/m-TiO_2_/perovskite/Spiro-OMeTAD/Au (Figure 5d), with and without the MK dye in the precursor solution. In order to maintain consistency, the same recipe for the precursor solution, as that of the mesoscopic solar cells, was employed. Interestingly, and as shown in Figure S14, with 3 mM of MK in the perovskite precursor solution, the resulting solar cells display much worse *J*–*V* performance, as compared with the control device based on pure MAPI. This suggests that the optimal additive concentration is not necessarily the same for the mesoscopic solar cells as for the thin-film n-i-p devices. Unfortunately, there are limited comparative studies across different device configurations of perovskite solar cells. However, similar observations can still be extracted from separate references employing 5-ammonium acid iodide (5-AVAI) as a precursor additive in PSCs.^45,46^ The optimal concentration of 5-AVAI is around 3−5 mol % for mesoscopic PSCs, but this concentration leads to a decrease in the PCE of fresh, standard thin-film solar cells despite an observed improved stability. Different crystallization processes and final morphologies of crystals in different device configurations may constitute possible reasons. Although this is not the focus of the present work, we still believe that it is an interesting observation that can help to guide further related studies and consequent discussion.

Hole-only devices were constructed with the configuration ITO/PEDOT:PSS/perovskite (with or without MK)/Spiro-OMeTAD/Au and the corresponding *I*–*V* characteristics in the dark are shown in Figure S15. Although based on a nonoptimized concentration, adding MK into the precursor solution resulted in a perovskite film with reduced defect density, as indicated by the lower trap-filled limit voltage (*V*_TFL_), which reflects trap states filling at a lower bias. A hole-only device without any perovskite included with the aim of investigating the hole mobility of MK as a standalone layer was constructed. However, the low MK solubility made it difficult to deposit uniform films through solution-processing techniques. This also poses a challenge to discuss the application of the MK dye as a hole-transport material, despite its favorable energetics for hole transfer discussed above. Nevertheless, the MK dye may prove a suitable dopant in existing hole-transport material systems or at the interface between perovskite and hole-transport layer, an active area of research today, often referred to as interface engineering.

More selectively, the performance of the MK dye at the perovskite/hole transport layer interface, i.e., as a post-treatment material, was investigated with a thin-film device configuration (Figure 5d) under 1-sun illumination. Instead of adding the MK dye into the precursor solution, an MK solution in IPA at different concentrations was spin-coated onto perovskite films. The *J*–*V* performance of fresh cells is shown in Figures 5c and S16. Compared with intrinsic MAPI, MK-coated samples show a gradual reduction of the average PCE for fresh samples, although the champion device formed with 0.002 mg mL^−1^ MK in the post-treatment solution displays similar *J*–*V* performance as that of the control devices. After storage under ambient conditions (20 °C, 40−60% RH), the control devices exhibit a decline in PCE, whereas the MK-treated devices demonstrate a general enhancement in PCE (Figures 5c and S17). The average PCE of the control devices dropped 28% after 20 days. In contrast, the average PCE of MK-treated devices maintained 95% of the initial PCE when 0.002 mg mL^−1^ MK post-treatment solutions had been used and produced 112% and 107% performances for the devices based on the concentrations 0.02 mg mL^−1^ and 0.2 mg mL^−1^, respectively, relative to the freshly prepared devices. This is consistent with results from the manually fabricated mesoscopic solar cells. Taken together, these results suggest that the stability of the solar cells can be improved by integrating the MK dye either in the precursor solution or as a postcoating on the perovskite films. Based on the device trends, the efficiency appears to depend on the dye concentration and fabrication conditions. Low MK concentrations can maintain comparable efficiencies while enhancing stability, whereas higher concentrations may introduce competing carrier redistribution or nonradiative pathways that reduce the device efficiency.

## 3 Conclusions

In summary, we have developed a robot-assisted strategy to investigate the performance of classical dyes in solar cells with a focus on their emerging roles in perovskite solar cells. This automated and readily iterative synthesis-characterization workflow standardizes the investigation process across systems comprising numerous candidate materials, including, but not limited to, dyes. The dye for demonstration, MK, has shown clear PL quenching behavior that motivated further analysis. The combination of experimental and theoretical studies shows that MK interacts with the perovskite surface or defect sites, modifies the interfacial electronic structure, and improves the stability of the dye-incorporated perovskite samples. Robotized fabrication and evaluation of mesoscopic PSCs also suggest a suitable range of dye additive concentrations in the precursor solutions, which was verified by manually fabricated devices. Although the initial PCE still requires further optimization, the manually fabricated PSCs containing the MK dye exhibit improved stability in different cell configurations. It is clear that different dye molecules may exert diverse effects on perovskite films and devices, including sensitization, defect passivation, carrier redistribution and long-term stabilization. For the currently studied dye, MK, the three latter effects dominate rather than sensitization, underscoring the importance of systematically exploring dye-perovskite interactions using automated approaches. Future efforts will focus on optimizing the process, improving the transferability of the robot-selected conditions and expanding automated studies to a broader range of functional molecules and perovskite materials. We believe this work may also inspire further research into different novel roles of dyes in perovskite solar cells and other related fields.

## 4 Experimental Section

### 4.1 Materials, Instrumentation, and Robotic Platform

All reagents were purchased from commercial sources and used as received without further purification or treatment unless otherwise stated. Detailed descriptions of the materials, instrumentation, and robotic platform are provided in the Supporting Experimental Details in the Supporting Information.

### 4.2 Synthesis-Characterization Workflow

#### 4.2.1 Preparation of ZrO_2_ Array

ZrO_2_ array used for the synthesis-characterization workflow was prepared by blade-coating ZrO_2_ paste on FTO substrate with a tape mask. The pattern was designed according to a 24-well plate so that the parameters for PL measurement in the plate reader can be set based on the default 24-well plate options. Four coordinates at the center (B3, B4, C3, C4) were removed for better vacuum gripping, leaving each substrate with 20 ZrO_2_ films of 1.6 cm in diameter. After the coating and mask removing, the substrate was heated at 400 °C for 40 min to remove the organic binders in the paste.

#### 4.2.2 Preparation of Solutions

For additive workflows, 0.6 M MAPbI_3_ (MAPI) precursor solution was prepared by mixing PbI_2_ and MAI in DMF. Dye stock solutions were prepared at 0.5 mM in DMF. For post-treatment workflow, MAPI precursor solution was prepared at 0.3 M in DMF, and MK solution was prepared at 1 mM in IPA.

#### 4.2.3 Automated Workflows

**Additive workflow**: robotic synthesis was designed to mix MAPI precursor solution with different concentrations of dye solutions. The synthesis starts with dispensing different volumes of DMF and dye stock solutions to different tubes. To each tube, 100 μL of MAPI solution was added, followed by a customized mixing procedure consisting of 10 cycles of slow aspiring and fast dispensing of 70% of the solution. Details for different dye solutions prepared in additive workflows are shown in Tables S1 and S2.

After solution preparation, 1 μL of each solution was dispensed on different ZrO_2_ substrates. Two samples were prepared for each condition. The 20 films on one substrate were heated together at 70 °C for 5 min, then transferred to a metal stage to cool to room temperature and sent to the plate reader for PL measurements.

**Post-treatment workflow**: MAPI films were prepared by dispensing 1 μL MAPI (0.3 M) on each ZrO_2_ substrate, followed by the same heating, cooling, and transferring procedure described above. After PL measurements, the substrate was transferred back to the liquid handling robot. Post-treatment solutions were prepared as shown in Table S3.

Ten μL of each post-treatment solution including the MK/IPA stock solution and pure solvent IPA and EA were dispensed on each ZrO_2_-MAPI film. Two samples were prepared for each condition, leaving two ZrO_2_-MAPI films untreated as the reference. After dispensing, the substrate was left at room temperature for 30 s to allow the solutions to spread and the solvent to evaporate. Afterward, the substrate was transferred back to the plate reader for PL measurements.

PL measurements were performed automatically with TECAN Infinite M Plex with a presaved method. The PL signal excited by 450 nm is collected from 500 to 850 nm with 1 nm step. In post-treatment workflow, kinetic PL cycles were investigated by setting 10 kinetic cycles with 15 min interval.

### 4.3 Additional Characterization

After the automatic workflows, the resulting FTO/ZrO_2_/perovskite­(/dye) array was cut into individual samples for subsequent investigation. Powder X-ray diffraction (PXRD) patterns were collected using a PANalytical-X’Pert PRO diffractometer with Cu−Kα radiation, operated at 45 kV and 40 mA. UV−vis Diffuse Reflectance Spectroscopy measurements were carried out with an Avantes AvaSpec-2048 dual UV−vis spectrometer equipped with an integrating sphere with an integrated light source. Water contact angle was determined using a PGX Contact Angle Goniometer.

Detailed experimental and computational characterization procedures for the dye, as well as additional thin-film fabrication and characterization details, are provided in the Suppporting Experimental Details in the Supporting Information.

### 4.4 Robotic Solar Cell Fabrication and Evaluation

The robotic solar cell fabrication and evaluation workflow followed our previously reported procedure,^32^ where detailed descriptions of the module setup, control methods, and substrate preparation are provided.

The printed mesoscopic solar-cell arrays contain 16 inorganic scaffolds on each substrate. Two μL of different solutions were dispensed on different inorganic scaffolds (Table S8). The robot arm transferred the substrate between the hot plate, cooling stage and solar cell module for 3 cycles. In each cycle, the substrate containing 16 solar cells was heated at 90 °C for 5 min, cooled to 30 °C, placed on the metal stage for additional 60 s and measured in the solar cell module under 1000 lx LED illumination (250.8 μW cm^−2^).^47^

### 4.5 Manual Device Fabrication and Characterization

#### 4.5.1 Carbon-Based Mesoscopic Solar Cells

Manual fabrication of carbon-based mesoscopic solar cells was based on commercially available monolithic perovskite solar cell electrodes (Solaronix). The architecture of the commercial electrodes is the same as our printed electrodes, which is FTO/cTiO_2_/mTiO_2_/ZrO_2_/Carbon, but the active area size is larger (12.5 mm × 12 mm). The electrodes were heated to 400 °C for 30 min and masked by Kapton tape before use. MAPI solutions with or without 3 mM MK were taken from robotic synthesis (Table S8). Each electrode was filled with 5 μL precursor solution and then heated at 70 °C for 30 min in ambient air (20 °C, 50% RH) and covered with a glass Petri dish.

#### 4.5.2 Thin-Film n-i-p Solar Cells

FTO was etched by Zn powder/2 M HCl solution and cleaned by sonication as described above. A compact TiO_2_ layer was deposited by spray pyrolysis of 0.2 M Ti­(acac)_2_OiPr_2_ solution in IPA at 450 °C. After cooling to room temperature, the substrate was cut into 1.5 cm × 2 cm pieces. A thin layer of mesoporous TiO_2_ was deposited by spin-coating diluted TiO_2_ paste (Solaronix, T/SP) at 4000 rpm for 15 s, followed by a stepwise annealing at 125 °C for 5 min, 325 °C for 5 min, 375 °C for 5 min and 450 °C for 30 min. The ratio of TiO_2_ paste and ethanol was 1:5.26 by weight. The mesoporous TiO_2_ layer was then doped with lithium via spin-coating (3000 rpm, 10 s) 10 mg mL^−1^ of Li-TFSI solution in acetonitrile and annealed with the same stepwise program. The perovskite films were deposited as described in the section of thin film preparation and characterization. For MK incorporated devices, MK was added to the precursor solution at a concentration of 3 mM, or MK solution in IPA with different concentrations (0.2 mg mL^−1^, 0.02 mg mL^−1^, 0.002 mg mL^−1^) was spin coated on perovskite films at 4000 rpm for 30 s. PEAI in IPA (2.5 mg mL^−1^) was spin coated at 4000 rpm for 30 s for all devices. The hole-transport layer was then deposited by spin-coating (4000 rpm, 30 s) Spiro-OMeTAD solution, which consisted of 90 mg Spiro-OMeTAD, 36 μL 4-tertbutylpyridine, 22 μL Li-TFSI stock solution (520 mg mL^−1^ in acetonitrile) and 1 mL chlorobenzene. Finally, 80 nm of Au was thermally evaporated using a shadow mask. Solutions were prepared in a glovebox, while all other steps were performed under ambient conditions (20 °C, 30−50% RH).

#### 4.5.3 Solar Cell Characterization

The *J*–*V* characteristics of manually fabricated solar cells were collected using a Keithley 2400 Source Meter under simulated 1-sun AM 1.5G illumination (Newport 91160−1000), which was calibrated to 100 mW cm^−2^ using a Si diode. The measurements were carried out in ambient air. A metal mask with aperture size of 0.1256 cm^−2^ was used. The devices were investigated in reverse scan mode (near *V*_OC_ to 0) with a step of 0.012 V and scan rate of 54.5 mV s^−1^. For each comparison, all cells were prepared in a single batch to ensure consistency and stored under ambient conditions together (20 °C, 40−60% RH).

#### 4.5.4 Hole-Only Device Fabrication and Characterization

To fabricate the hole-only device, ITO was etched and cleaned following the procedure for FTO described above. PEDOT:PSS (Ossila) was spin-coated at 4000 rpm for 30 s, followed by thermal treatment at 135 °C for 30 min. After cooling to room temperature, the substrates were UV-Ozone treated for 15 min before the next step. The deposition of perovskite (with or without 3 mM MK), Spiro-OMeTAD and Au layers was the same as for the solar cell device fabrication. *I*–*V* characterization of the hole-only devices were performed in the dark using a Keithley 2400 Source Meter.

## Supplementary Material





## Notes

checkCIF MK245 (PDF)
Experimental information: detailed descriptions of the materials, instrumentation, robotic platform, dye and single-crystal characterization, thin-film preparation and characterization, and computational details; Figures and Tables: images of the robotic synthesis, images of the robotic platform and printed substrate; comparison of PL changes with different dye additives; supplementary PXRD patterns of samples from robotic workflows and extracted fwhm data; PXRD of printed substrates; UV−vis DRS of samples from the additive workflow; molecular structure, ORTEP representation, crystal structure determination data, simulated PXRD and additional DFT results of the MK dye; details of the robotized solar-cell evaluation workflow with original *I*−*V* curves; IPCE of manually fabricated mesoscopic cells; *I*−*V* curves of hole-only devices; and additional device performance of manually fabricated solar cells; Data: Photovoltaic performance metrics of robotically and manually fabricated solar cells (PDF)
