# Supplementary material for: Robot-Assisted Study of a Near-Infrared Dye in Perovskite Solar Cells
Source: ACS Appl Mater Interfaces. 2026 Jun 30;18(27):37628–38. doi: 10.1021/acsami.6c05093 (PMC13383275; doi:10.1021/acsami.6c05093)

checkCIF/PLATON report

You have not supplied any structure factors. As a result the full set of tests cannot be run.

THIS REPORT IS FOR GUIDANCE ONLY. IF USED AS PART OF A REVIEW PROCEDURE FOR PUBLICATION, IT SHOULD NOT REPLACE THE EXPERTISE OF AN EXPERIENCED CRYSTALLOGRAPHIC REFEREE.

No syntax errors found.   CIF dictionary   Interpreting this report

Datablock: orto_b

```
Bond precision:   C-C = 0.0078 A          Wavelength=0.61992

Cell:               a=7.9900(16)       b=35.116(7)       c=26.375(5)
                    alpha=90           beta=90           gamma=90
Temperature:        100 K

                 Calculated                     Reported
Volume           7400(3)                        7400(3)
Space group      P c a 21                       P c a 21
Hall group       P 2c -2ac                      P 2c -2ac
Moiety formula   C44 H44 Cl N2 O4, Cl           C44 H44 Cl N2 O4, Cl
Sum formula      C44 H44 Cl2 N2 O4              C44 H44 Cl2 N2 O4
Mr               735.71                         735.71
Dx,g cm-3        1.321                          1.321
Z                8                              8
Mu (mm-1)        0.155                          0.155
F000             3104.0                         3104.0
F000'            3106.54
h,k,lmax         11,51,38                       11,48,36
Nref             24720[ 12610]                  23526
Tmin,Tmax        0.994,0.997                    0.790,0.868
Tmin'            0.991

Correction method= # Reported T Limits: Tmin=0.790 Tmax=0.868
AbsCorr = EMPIRICAL

Data completeness= 1.87/0.95       Theta(max)= 27.150

                                                     wR2(reflections)=
R(reflections)= 0.0847( 19362)                       0.2583( 23526)

S = 1.067                    Npar= 949
```

The following ALERTS were generated. Each ALERT has the format
test-name_ALERT_alert-type_alert-level.
Click on the hyperlinks for more details of the test.

Alert level B

```
PLAT230_ALERT_2_B Hirshfeld Test Diff for    C4       --C5       .        7.2 s.u.
PLAT230_ALERT_2_B Hirshfeld Test Diff for    C12      --C69      .        9.5 s.u.
PLAT230_ALERT_2_B Hirshfeld Test Diff for    C48      --C49      .        8.2 s.u.
PLAT230_ALERT_2_B Hirshfeld Test Diff for    C67      --C68      .        9.7 s.u.
PLAT230_ALERT_2_B Hirshfeld Test Diff for    C72      --C73      .        9.7 s.u.
```

Alert level C

```
STRVA01_ALERT_4_C           Flack test results are ambiguous.
          From the CIF: _refine_ls_abs_structure_Flack    0.495
          From the CIF: _refine_ls_abs_structure_Flack_su    0.005
PLAT084_ALERT_3_C High wR2 Value (i.e. > 0.25) ...................       0.26 Report
PLAT230_ALERT_2_C Hirshfeld Test Diff for    C7       --C8       .        6.0 s.u.
PLAT230_ALERT_2_C Hirshfeld Test Diff for    C34      --C35      .        6.0 s.u.
PLAT230_ALERT_2_C Hirshfeld Test Diff for    C55      --C58      .        7.0 s.u.
PLAT230_ALERT_2_C Hirshfeld Test Diff for    C74      --C75      .        5.7 s.u.
PLAT340_ALERT_3_C Low Bond Precision on  C-C Bonds ...............    0.00782 Ang.
```

Alert level G

```
ABSMU01_ALERT_1_G  Calculation of _exptl_absorpt_correction_mu
                not performed for this radiation type.
PLAT007_ALERT_5_G Number of Unrefined Donor-H Atoms .............          4 Report
              H2    H3    H6    H8
PLAT012_ALERT_1_G N.O.K.   _shelx_res_checksum Found in CIF ......     Please Check
PLAT111_ALERT_2_G ADDSYM Detects New (Pseudo) Centre of Symmetry .        100 %Fit
PLAT112_ALERT_2_G ADDSYM Detects New (Pseudo) Symm. Elem         b        100 %Fit
PLAT113_ALERT_2_G ADDSYM Suggests Possible Pseudo/New  Space Group       Pbca Check
              Check Model Parameter Symmetry for Reflection Data Support
PLAT335_ALERT_2_G Check Large C6 Ring C-C Range C45      -C50            0.17 Ang.
PLAT802_ALERT_4_G CIF Input Record(s) with more than 80 Characters          2 Info
PLAT883_ALERT_1_G Absent Datum for _atom_sites_solution_primary ..     Please Do !
PLAT899_ALERT_4_G SHELXL2018 is Outdated   and Succeeded by SHELXL     2019/3 Note
PLAT951_ALERT_5_G Calculated (ThMax) and CIF-Reported Kmax Differ           3 Units
PLAT952_ALERT_5_G Calculated (ThMax) and CIF-Reported Lmax Differ.          2 Units
PLAT965_ALERT_2_G The SHELXL WEIGHT Optimisation has not Converged     Please Check
PLAT967_ALERT_5_G Note: Two-Theta Cutoff Value in Embedded .res ..       55.0 Degree
PLAT984_ALERT_1_G The  C-f'=    0.1200 Deviates from the B&C-Value     0.0020 Check
PLAT984_ALERT_1_G The Cl-f'=    2.4000 Deviates from the B&C-Value     0.1198 Check
PLAT984_ALERT_1_G The  N-f'=    0.1000 Deviates from the B&C-Value     0.0042 Check
PLAT984_ALERT_1_G The  O-f'=    0.1500 Deviates from the B&C-Value     0.0077 Check
PLAT985_ALERT_1_G The  C-f"=    0.0500 Deviates from the B&C-Value     0.0012 Check
PLAT985_ALERT_1_G The Cl-f"=    1.1000 Deviates from the B&C-Value     0.1215 Check
PLAT985_ALERT_1_G The  N-f"=    0.0300 Deviates from the B&C-Value     0.0024 Check
PLAT985_ALERT_1_G The  O-f"=    0.0400 Deviates from the B&C-Value     0.0045 Check
```

---

0 **ALERT level A** = Most likely a serious problem - resolve or explain
5 **ALERT level B** = A potentially serious problem, consider carefully

```
 7 ALERT level C = Check. Ensure it is not caused by an omission or oversight
22 ALERT level G = General information/check it is not something unexpected

11 ALERT type 1 CIF construction/syntax error, inconsistent or missing data
14 ALERT type 2 Indicator that the structure model may be wrong or deficient
 2 ALERT type 3 Indicator that the structure quality may be low
 3 ALERT type 4 Improvement, methodology, query or suggestion
 4 ALERT type 5 Informative message, check
```

It is advisable to attempt to resolve as many as possible of the alerts in all categories. Often the minor alerts point to easily fixed oversights, errors and omissions in your CIF or refinement strategy, so attention to these fine details can be worthwhile. In order to resolve some of the more serious problems it may be necessary to carry out additional measurements or structure refinements. However, the purpose of your study may justify the reported deviations and the more serious of these should normally be commented upon in the discussion or experimental section of a paper or in the "special_details" fields of the CIF. checkCIF was carefully designed to identify outliers and unusual parameters, but every test has its limitations and alerts that are not important in a particular case may appear. Conversely, the absence of alerts does not guarantee there are no aspects of the results needing attention. It is up to the individual to critically assess their own results and, if necessary, seek expert advice.

Publication of your CIF in IUCr journals

A basic structural check has been run on your CIF. These basic checks will be run on all CIFs submitted for publication in IUCr journals (*Acta Crystallographica*, *Journal of Applied Crystallography*, *Journal of Synchrotron Radiation*); however, if you intend to submit to *Acta Crystallographica Section C* or *E* or *IUCrData*, you should make sure that full publication checks are run on the final version of your CIF prior to submission.

Publication of your CIF in other journals

Please refer to the *Notes for Authors* of the relevant journal for any special instructions relating to CIF submission.

PLATON version of 04/06/2025; check.def file version of 30/05/2025

Datablock orto_b - ellipsoid plot

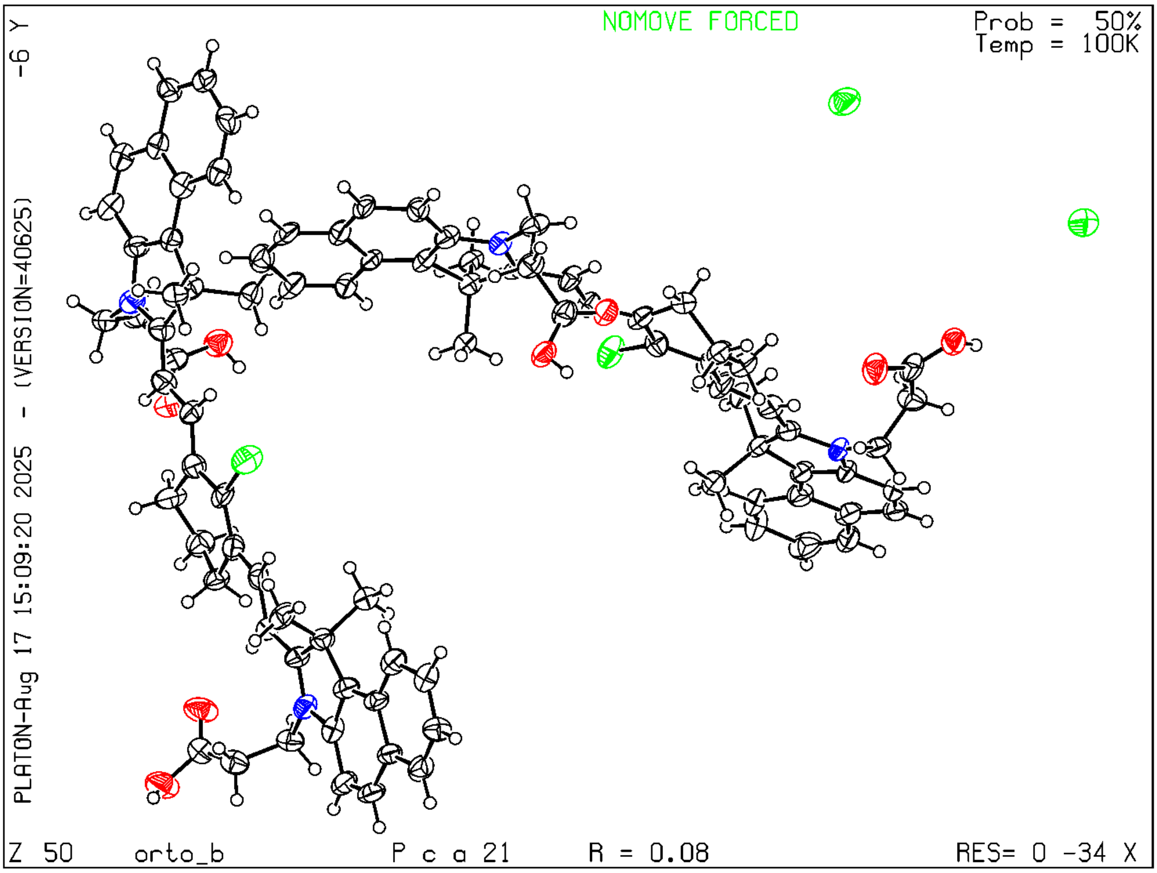

Supplement: Supplementary file 1 [file am6c05093_si_001.pdf]
