# Supplementary material for: Robot-Assisted Study of a Near-Infrared Dye in Perovskite Solar Cells
Source: ACS Appl Mater Interfaces. 2026 Jun 30;18(27):37628–38. doi: 10.1021/acsami.6c05093 (PMC13383275; doi:10.1021/acsami.6c05093)
Supplement: Supplementary file 2 [file am6c05093_si_002.pdf]

Supporting Information

Robot-Assisted Study of a Near-Infrared Dye in Perovskite Solar Cells

Bingyu Lei[1], Per H. Svensson[1], Lars Kloo[1,]*

[1]Applied Physical Chemistry, Department of Chemistry, KTH Royal Institute of Technology, Stockholm, SE-114 28 Stockholm, Sweden

Correspondence Email: lakloo@kth.se

Supplementary Experimental Details

Materials:

Laser patterned glass substrates with a conducting layer of fluorine-doped tin oxide (FTO) of 7 Ω/sq sheet resistance were purchased from Yingkou Shangneng Photoelectric Material Co., Ltd. Unpatterned FTO (15 Ω/sq) was purchased from Hartford Glass Co., Inc. Methylammonium iodide (MAI) was purchased from Greatcell Solar Ltd. Lead iodide, Quinaldine Red (QR) and Indigo were purchased from TCI CO. Titanium diisopropoxide bis(acetylacetonate) ($Ti(acac)_2OiPr_2$), Spiro-OMeTAD (SHT-263S), Phenethylammonium iodide (PEAI), Bis(trifluoromethane)sulfonimide lithium salt (Li-TFSI), 4-tert-butylpyridine, *N, N*-dimethylformamide (DMF, anhydrous), dimethyl sulfoxide (DMSO, anhydrous), isopropanol (IPA, anhydrous), ethyl acetate (anhydrous), chlorobenzene(anhydrous), acetonitrile(anhydrous) and RBS 25 solution were purchased from Merck. Isopropanol and acetone were purchased from VWR. TiO_2 (T165), ZrO_2 and carbon pastes were purchased from Solaronix. Victorian Blue B (VBB) was purchased from Santa Cruz Biotechnology. MK245 was obtained from the manufacturer. All reagents were used as received without further purification or treatment unless otherwise stated.

Instrumentation:

Liquid handling robot: Opentrons OT-2 pipetting robot

Robot arm: Ufactory Xarm 6 integrated with Ufactory vacuum gripper

Plate reader: TECAN Infinite M Plex

A custom-made multichannel potentiostat assembly (designed by P&L Scientific AB, Sweden) with EmStat4S potentiostat (Palmsens. Inc.)

Robotic platform:

The robotic platform used in this work is based on the previously reported AURORA system.[1] Details about the solar cell fabrication and evaluation modules and workflows are available in previously published work.

For the synthesis-characterization workflow, the liquid handling robot and robot arm were connected to the control laptop through a local area network, and the plate reader was connected via USB. The corresponding orchestrator Python script contains:

1) liquid-handling robot protocol handler to send protocol for solution preparation and film deposition
2) status check function to make sure robot arm moving into liquid-handling robot after the liquid-handling robot finish running
3) different robot arm functions for different movements
4) subprocess command to invoke pre-saved Magellan™ methods for photoluminescence (PL) measurements
5) PyAutoGUI commands to interact with Magellan™ software, such as starting the measurement and saving the data

The liquid-handling protocol handler was developed based on Opentrons integration tools using Opentrons HTTP API (https://github.com/Opentrons/opentrons-integration-tools/tree/main/http-api). The robotic arm functions were developed based on the Python script generated by training the robotic arm via Ufactory Blocky.

MK245 characterization

Optical characterization

The MK245 solution prepared from the post-treatment workflow was used to determine the UV-vis absorption. 50 µL of the MK solution (0.1 mM in IPA) was transferred to a microplate well and measured with TECAN Infinite M Plex. Due to the limited wavelength range of the plate reader, the PL of the MK245 solution was recorded using a Cary Eclipse fluorescence spectrometer with an excitation wavelength of 800 nm.

Single crystal preparation and measurement

Single crystals of MK245 were collected using the anti-solvent vapor diffusion method. More specifically, a vial containing MK245/DMF solution was placed inside a larger sealed vial containing ethyl acetate. Over time, diffusion of the anti-solvent into the dye solution promoted slow crystallization.

The diffraction data were collected on a Synchrotron radiation ($\lambda = 0.61992$ Å). The diffraction data was collected at ESRF beamline ID23 using the MXCuBE data-acquisition interface, and the data were integrated with XDS. The structure was solved with direct methods (SHELXS-2015) and refined using full-matrix least-squares based on F2 with the programs SHELXS-97 and SHELXL-97. All nonhydrogen atoms were refined anisotropically, and the hydrogen atoms were included at idealized positions. The crystal structure is visualized by Figure S9. Detailed information on the crystal data, data collection, and refinement data is provided in Table S6.

Thin-film preparation and characterization

FTO or glass was cut into pieces of size 1.5 cm × 2 cm and was sonicated in RBS 25 water solution, isopropanol and acetone for 15 minutes sequentially. The substrates were dried in air and then treated with UV-Ozone for 10 minutes prior to thin-film preparation.

MAPI solution was prepared by dissolving 0.461g PbI_2 and 0.159 g MAI in 630 μL DMF and 70 μL DMSO. After stirring at room temperature overnight, the solution was filtered using a 200 nm PTFE syringe filter.

To prepare MAPI thin films, 40 μL of precursor solution was spin-coated on substrate by a three-step spin-coating procedure, namely 500 rpm for 5 seconds, 1000 rpm for 10 seconds and 5000 rpm for 25 seconds. 800 μL of anti-solvent was dripped 8 seconds before the end of the last step, followed by thermal annealing at 100 °C for 5 min under ambient air conditions. MK245 was dissolved in IPA to a concentration of 0.02 mg mL^{-1} and was spin-coated onto the MAPI film at a spin rate of 4000 rpm for 30 seconds.

Samples prepared on glass were used for PXRD measurements, while those on FTO were used for X-ray photoelectron spectroscopy (XPS) and ultraviolet photoelectron spectroscopy (UPS) using a Kratos AXIS Supra+ X-ray photoelectron spectrometer, as well as for scanning electron microscopy (SEM), studied by Thermo Fisher Scientific Apreo 2s LoVac scanning electron microscope.

Computational details

The neutral (monoprotonated) dye molecule MK245 was modelled at the level of density-functional theory (DFT). The base functional used was the hybrid density functional B3LYP as implemented in Gaussian 16 (Rev. C.01).[2,3] The results of a series of functionals were investigated as a sensitivity test of the reliability of the excitation results emerging from the computations. The optical results were taken from the O3LYP functional, since it is frequently reported to provide relevant results with respect to experimental results. The results from all functionals studies are given in Table S7. The molecular structure was geometrically optimized. Single-point, time-dependent computations (TD-DFT) were performed on the geometrically optimized structures using the same density functional as for geometry optimization. 6-31G(d,p) basis sets were used for all the elements (H, C, N, O and Cl) in the geometry optimization, whereas in the TD-DFT computations the basis sets were expanded to 6-311G(p,d) quality. The following functionals were included in the sensitivity study: B3LYP,[3] cam- B3LYP,[4] B3PW91,[5] wB97xD,[6] M06-2X[7] and O3LYP[8]. Implicit solvent effects were modelled using the dielectric properties of ethanol in the polarizable continuum model (PCM).[9]

MK245 coordinated to the $FAPbI_3$ (FAPI) and MAPI systems were modelled using the hybrid density functional cam-B3LYP.[2,4] The MK245 was non-tethered to give the optimal coordination to the FAPI/MAPI slabs, which were kept frozen during the optimizations. 6-311+G(d,p) basis sets were used for all the lighter elements (H, C, N, O and Cl), and Stuttgart-Dresden-Cologne effective core potential-based basis sets with double-zeta quality valence space for I and Pb (MDF28 for I and MDF60 for Pb).[10–12]

Supplementary Figures and Tables

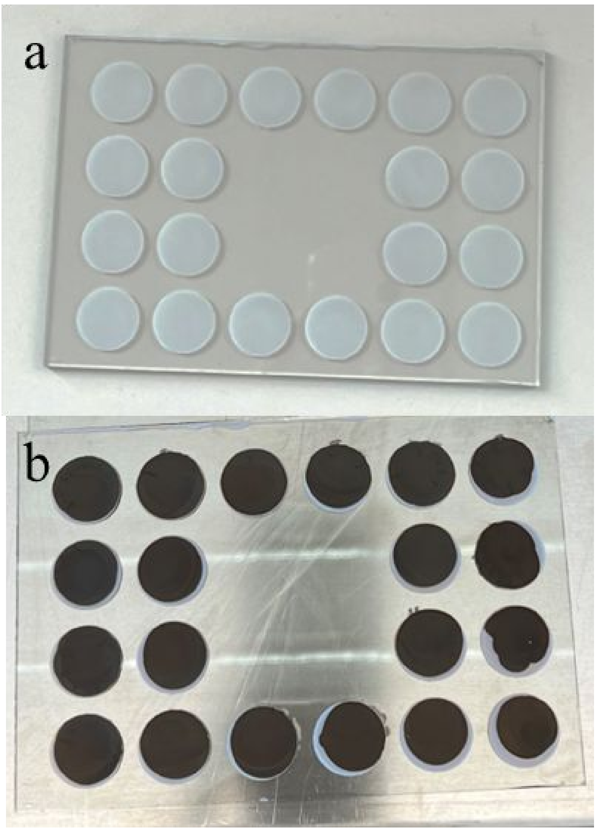

Figure S1. Images of the printed ZrO_2 array used in this work. (a) bare ZrO_2; (b) after perovskite deposition

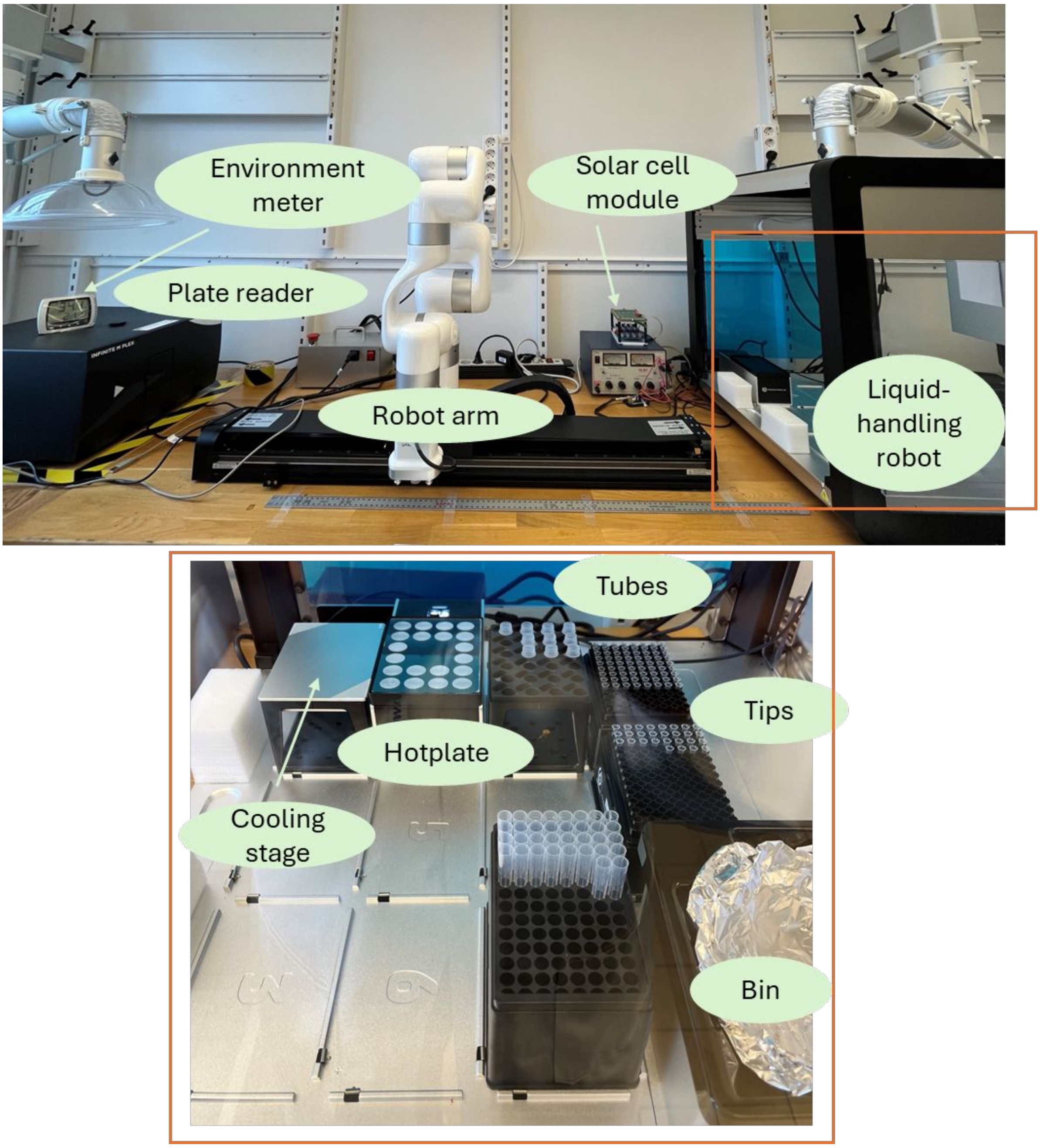

Figure S2. Image of the AURORA platform with modules used in this work.

Table S1. Details of robotic synthesis for studying MK245 as precursor additive for perovskite.

Tube	1	2	3	4	5	6	7	8	9	10
DMF (µL)	10	20	30	40	50	60	70	80	90	100
MK stock (µL)	90	80	70	60	50	40	30	20	10	0
MAPI stock (µL)	100	100	100	100	100	100	100	100	100	100
MK concentration (mM)	2.25×10^{-1}	2.00×10^{-1}	1.75×10^{-1}	1.50×10^{-1}	1.25×10^{-1}	1.00×10^{-1}	7.50×10^{-2}	5.00×10^{-2}	2.50×10^{-2}	0

Table S2. Details of robotic synthesis for studying other dyes as precursor additive for perovskite.

Dye	VBB			QR			Indigo			
**Tube**	**1**	**2**	**3**	**4**	**5**	**6**	**7**	**8**	**9**	**10**
DMF (µL)		50	90		50	90		50	90	100
Dye stock (µL)	100	50	10	100	50	10	100	50	10	0
MAPI stock (µL)	100	100	100	100	100	100	100	100	100	100
Dye concentration (mM)	2.50×10^{-1}	1.25×10^{-1}	2.50×10^{-2}	2.50×10^{-1}	1.25×10^{-1}	2.50×10^{-2}	2.50×10^{-1}	1.25×10^{-1}	2.50×10^{-2}	0

Table S3. Details of robotic synthesis for studying MK245 as post-treatment material for perovskite.

Tube	1	2	3	4	5	6
Solvent	IPA	EA	IPA	EA	IPA	EA
Volume (µL)	135	135	135	135	135	135
Parent solution	MK/IPA 1mM	MK/IPA 1mM	Tube 1	Tube 2	Tube 3	Tube 4
Volume (µL)	15	15	15	15	15	15
Dye concentration (mM)	1×10^{-1}	1×10^{-1}	1×10^{-2}	1×10^{-2}	1×10^{-3}	1×10^{-3}

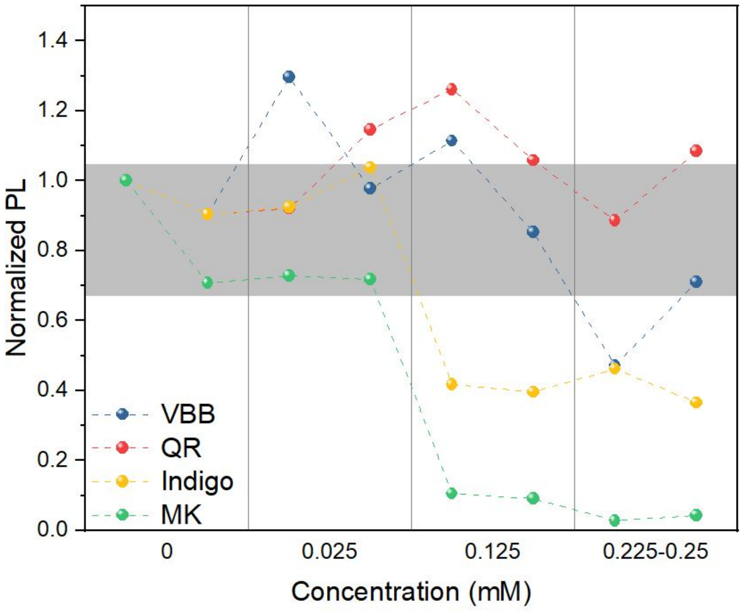

Figure S3. Comparison of the PL change after adding different amounts of dye to the perovskite precursor solution. VBB: Victorian Blue B, QR: Quinaldine Red, MK: MK245.

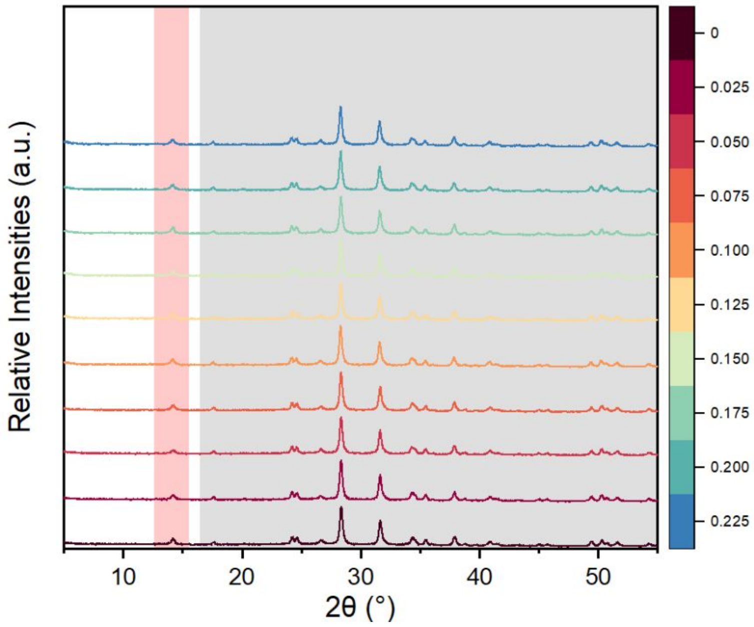

Figure S4. PXRD traces of robot-synthesized samples from the additive workflow. Labels denote the final concentrations (mM) of the MK245 in precursor solutions.

Table S4. FWHM of peaks located at 2θ = 14.2° for samples from the additive workflow.

Concentration of MK (mM)	FWHM
0	0.321
0.025	0.264
0.050	0.226
0.075	0.228
0.100	0.328
0.125	0.313
0.150	0.273
0.175	0.301
0.200	0.250
0.225	0.267

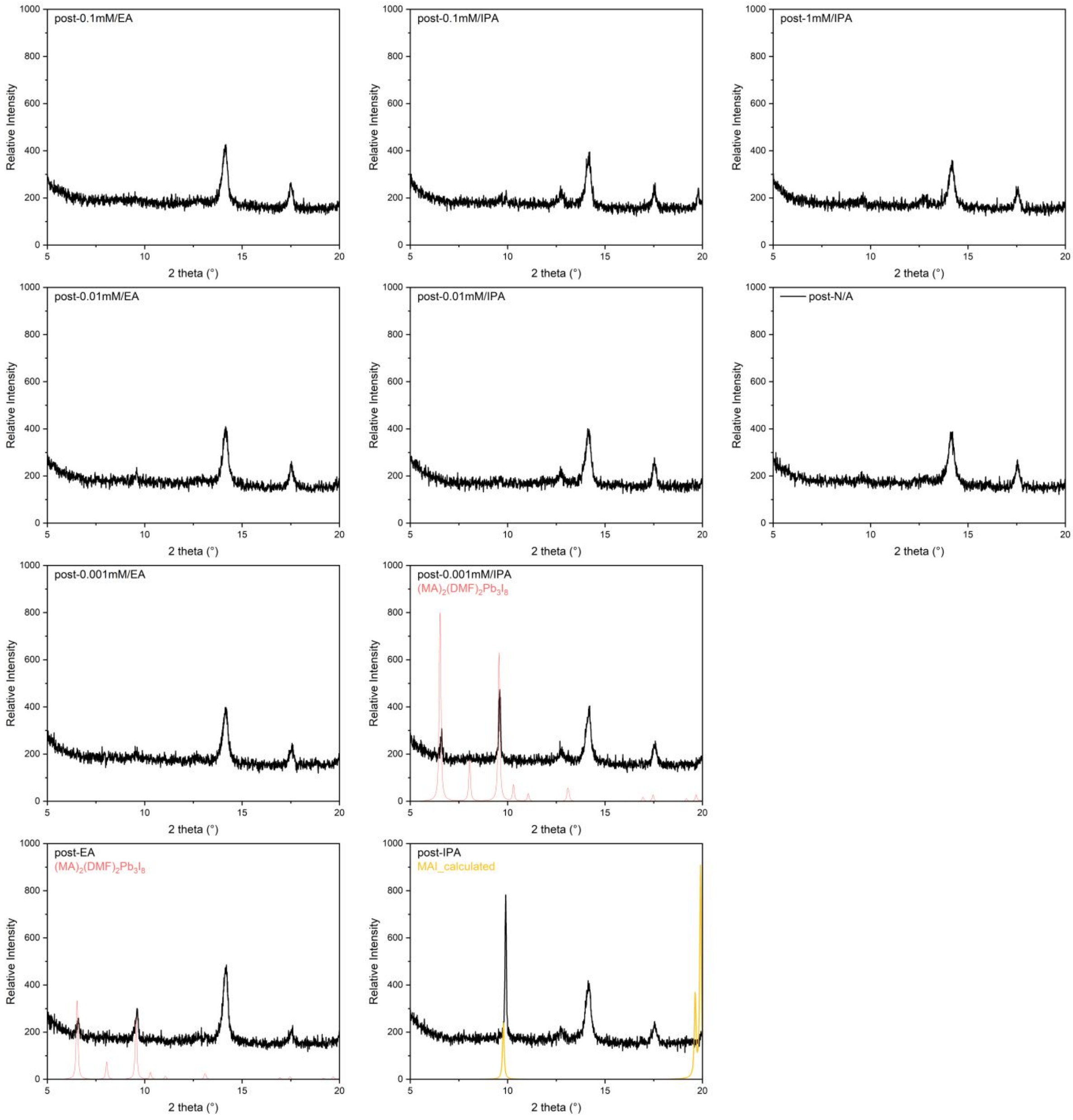

Figure S5. PXRD traces of robot-synthesized samples from the post-treatment workflow. Labels indicate the concentration of the MK245 in different solvents used for post-treatment solutions.

Table S5. FWHM of peaks located at 2θ = 14.2° for samples from the post-treatment workflow.

Solvent	Concentration of MK (mM)	FWHM
EA	0	0.275
EA	0.001	0.315
EA	0.01	0.297
EA	0.1	0.323
N/A	N/A	0.321
IPA	1	0.276
IPA	0.1	0.288
IPA	0.01	0.285
IPA	0.001	0.273
IPA	0	0.308

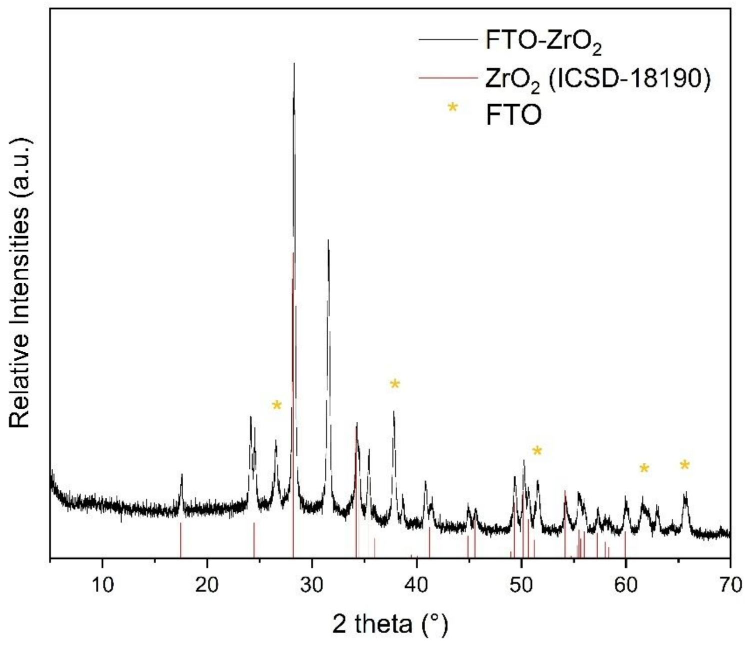

Figure S6. PXRD traces of printed ZrO_2 compared with reported PXRD data (ICSD-18190).

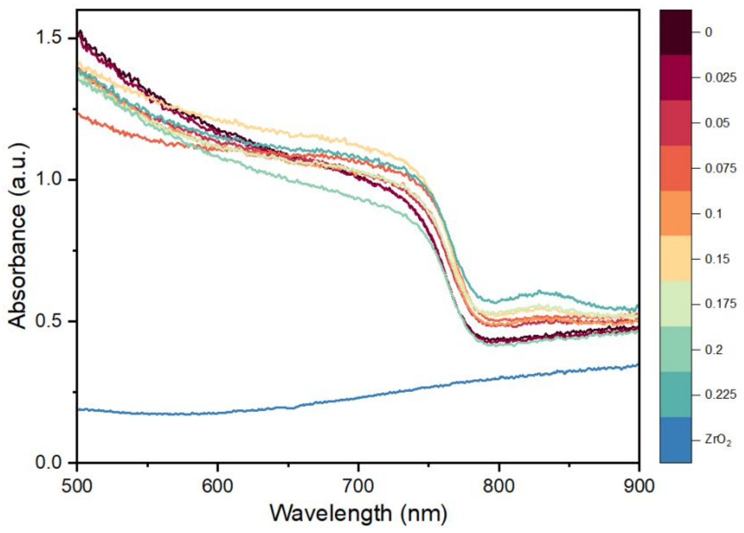

Figure S7. UV-Vis Diffuse Reflectance Spectroscopy results of robot-synthesized samples from the additive workflow. Labels denote the final concentrations (mM) of the MK245 in precursor solutions.

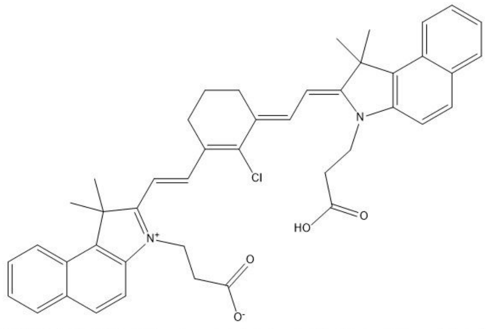

Figure S8. Molecular structure of the dye MK245.

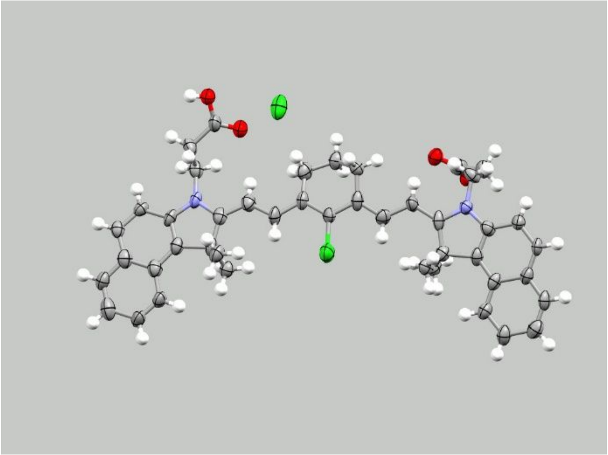

Figure S9. ORTEP representation of MK245 • HCl with 50% probability ellipsoids.

Table S6. Crystal and structure determination data

Identification code	MK245
CCDC number	2481131
Empirical formula	$C_{44}H_{44}Cl_2N_2O_4$
Formula weight	735.71
Temperature/K	100
Crystal system	Orthorhombic
Space group	P c a 21
a/Å	7.9900(16)
b/Å	35.116(7)
c/Å	26.375(5)
α/°	90
β/°	90
γ/°	90
Volume/Å^3	7400(3)
Z	8
ρ_{calc}g/cm^3	1.321
μ/mm^{-1}	0.155
F(000)	2944
Crystal size/mm^3	0.02 x 0.03 x 0.06
Radiation	Synchrotron, λ = 0.61992 Å
2*θ* range for data collection/°	2θ ≤ 54.3° (θ ≤ 27.150°)
Index ranges	-11 ≤ h ≤ 11, -48 ≤ k ≤ 48, -36 ≤ l ≤ 35
Reflections collected	137028
Independent reflections	23526
Data/restraints/parameters	Data = 23526, Restraints = 1, Parameters = 949
Goodness-of-fit on F^2	1.067
Final R indexes [I>=2σ (I)]	R = 0.0847
Final R indexes [all data]	R = 0.0969, wR2 = 0.2583
Largest diff. peak/hole / e Å^{-3}	0.787 / -1.182

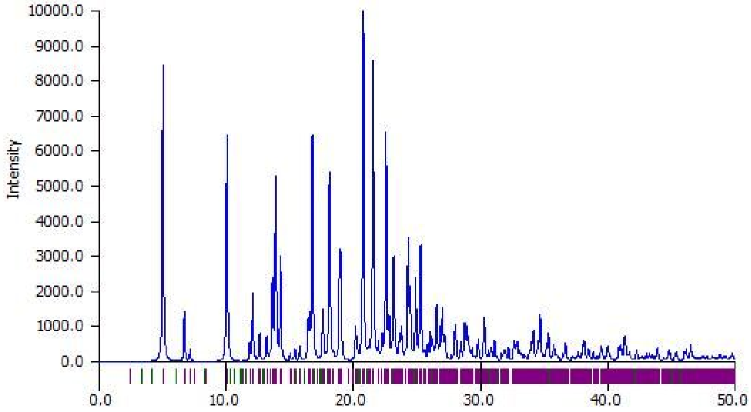

Figure S10. Simulated PXRD trace of the chloride salt of MK245.

Table S7. MK245 TD-DFT Results --- A sensitivity test based on a summary of results obtained from different density functionals

System	Property	cam-B3LYP	B3LYP	B3PW91	wB97xD	M06-2X	O3LYP
**MK245**	*1st str trans / nm; eV**	586.7; 2.11<br>*f* = 2.03 (root 2)<br>s→s **185->186**	623.2; 1.99<br>*f* = 1.55 (root 5)<br>s→s **184**,**185->186**	630.8; 1.97<br>*f* = 1.55 (root 3)<br>s→s **183-185->186**	583.0; 2.13<br>*f* = 1.99 (root 2)<br>s→s **185->186**	688.3; 1.80<br>*f* = 2.25 (root 2)<br>s→s **185->186**	646.1; 1.92<br>*f* = 0.96 (root 6)<br>s→s **181-185->186**
	*1st str trans / nm; eV (in EtOH)**	674.1; 1.84<br>*f* = 2.29 (root 2)<br>s→s **184**,**185->186**	753.2; 1.65<br>*F* = 2.38 (root 2)<br>s→s **185->186**	747.8; 1.66<br>*f* = 2.39 (root 2)<br>s→s **185->186**	619.3; 2.00<br>*f* = 2.21 (root 2)<br>s→s **184**,**185->186**	601.7; 2.06<br>*f* = 2.00 (root 2)<br>s→s **185->186**	780.6; 1.59<br>*f* = 2.30 (root 2)<br>s→s **185->186**
	HOMO / eV	-6.177	-5.231	-5.202	-6.636	-6.036	-4.683
	LUMO / eV	-2.213	-3.196	-3.161	-1.641	-2.476	-3.250
	E(tot) / H	-2572.18322309	-2573.76744241	-2572.46759330	-2572.64341439	-2572.39557761	-2572.53783064
	Dipole moment / D	11.33	11.55	11.43	10.99	10.14	12.18
**$MK245^-$**	*1st str trans / nm; eV**	589.11; 2.10,<br>*f* = 2.38 (root 2)<br>s→s **183**,**185->186**	616.8; 2.01<br>*f* = 2.29 (**root 15**)<br>s→s **179->186**	612.4; 2.02<br>*f* = 2.29 (**root 15**)<br>s→s **179->186**	585.4; 2.12<br>*f* = 2.43 (root 2)<br>s→s **183**,**185->186**	586.2; 2.12<br>*f* = 2.44 (**root 2**)<br>s→s **184**,**185->186**	639.5; 1.94<br>*f* = 1.48 (root 19)<br>s→s **179**,**182**,**184**,**185->186**
	*1st str trans / nm; eV (in EtOH)**	730.2; 1.70<br>*F* = 2.67 (root 2)<br>s→s **185->186**	759.7; 1.63<br>*f* = 2.69 (root 2)<br>s→s **185->186**	755.0; 1.64<br>*f* = 2.70 (root 2)<br>s→s **185->186**	731.7; 1.69<br>*f* = 2.65 (root 2)<br>s→s **185->186**	741.6; 1.67<br>*f* = 2.65 (root 2)<br>s→s **185->186**	779.8; 1.59<br>*f* = 2.64 (root 2)<br>s→s **185->186**
	HOMO / eV	-4.404	-2.881	-2.822	-4.863	-4.195	-2.260
	LUMO / eV	-0.588	-1.539	-1.495	-0.021	-0.713	-1.529
	E(tot) / H	-2571.63757614	-2573.22486702	-2571.92177176	-2571.09138280	-2571.84583976	-2571.99309926
	Dipole moment / D	25.02	24.73	25.22	24.37	18.30	24.69

*s→s denotes a singlet-to-singlet transition; for the all the MK245 molecules, MO no. 185 represents the HOMO and no. 186 the LUMO

Table S8. Details of solutions and devices in the robotized solar-cell evaluation workflow.

No. of solution	Concentration of MK (mM)	No. of solar cell on substrate 1	No. of solar cell on substrate 2	Label of cell
[0]	7	15, 16	1, 2	7-1, 7-2
[1]	6	13, 14	3, 4	6-1, 6-2
[2]	5	11, 12	5, 6	5-1, 5-2
[3]	4	9, 10	7, 8	4-1, 4-2
[4]	3	7, 8	9, 10	3-1, 3-2
[5]	2	5, 6	11, 12	2-1, 2-2
[6]	1	3, 4	13, 14	1-1, 1-2
[7]	0	1, 2	15, 16	0-1, 0-2

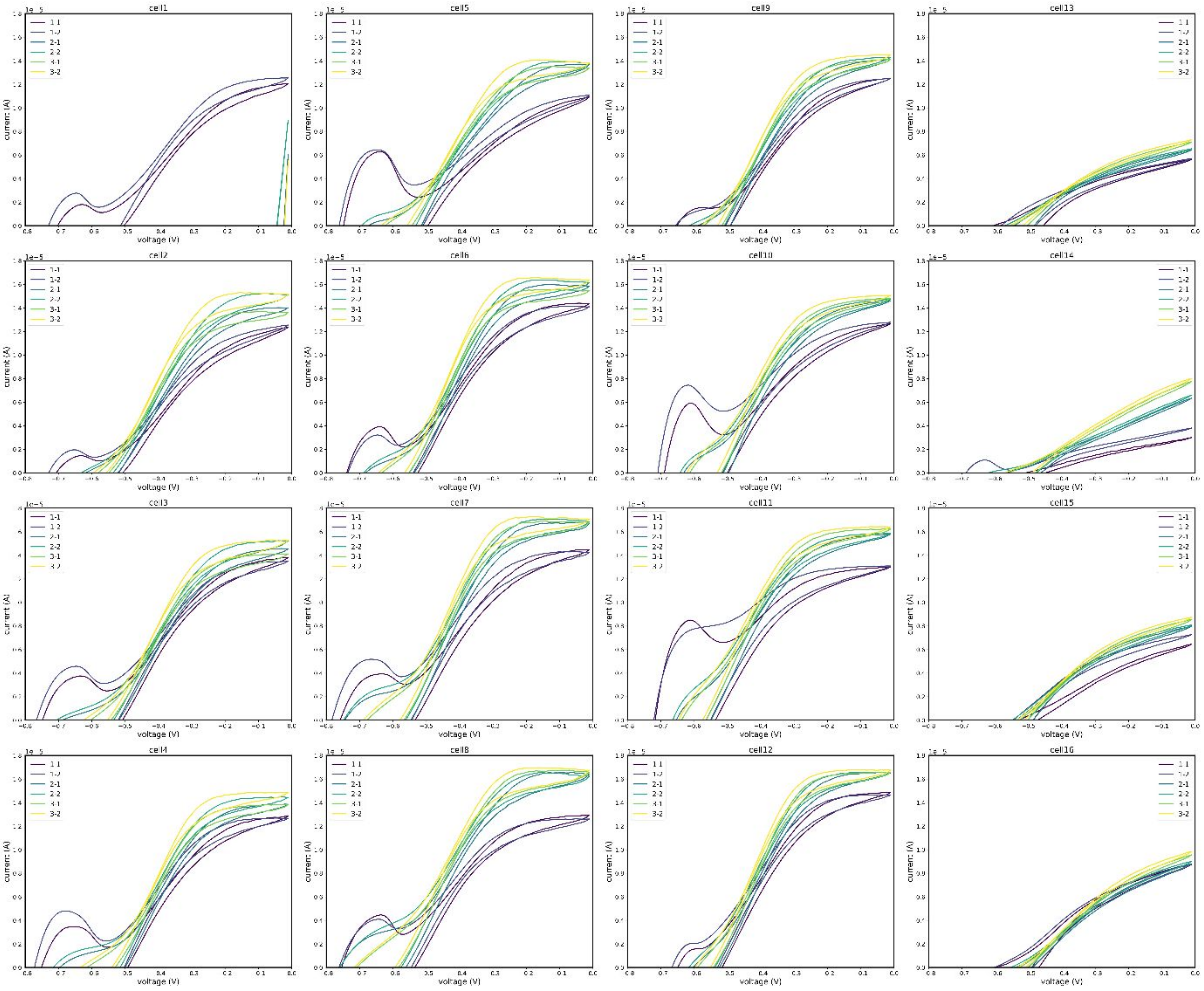

Figure S11. Original *I-V* curves of solar cells on substrate 1 from the robotic solar-cell fabrication-evaluation workflow. The labels (e.g., 1-1, 1-2, etc.) in each figure denote different cycle numbers and the number of measurements within each cycle.

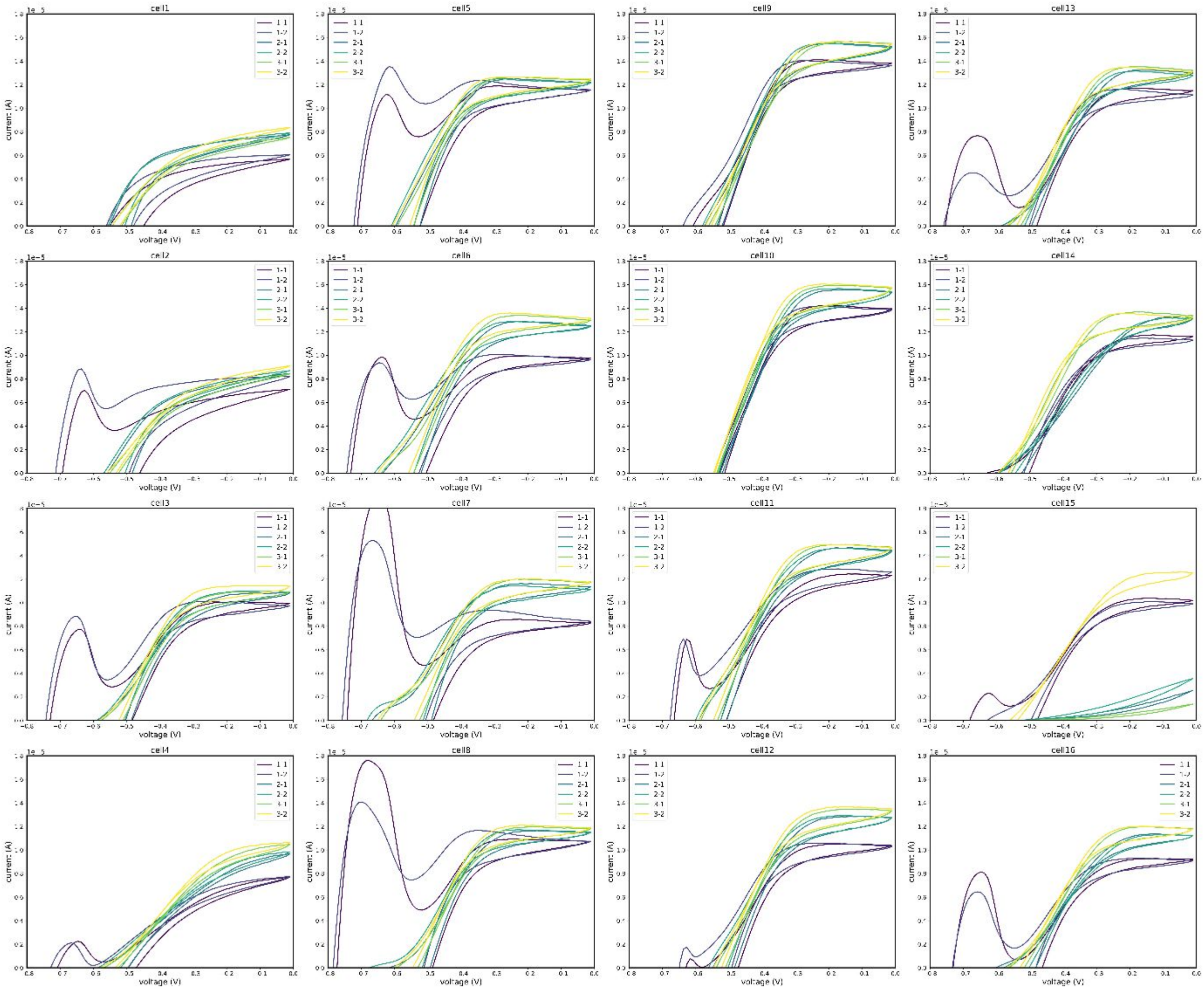

Figure S12. Original *I-V* curves of solar cells on substrate 2 from the robotic solar-cell fabrication-evaluation workflow. The labels (e.g., 1-1, 1-2, etc.) in each figure denote different cycle numbers and the number of measurements within each cycle.

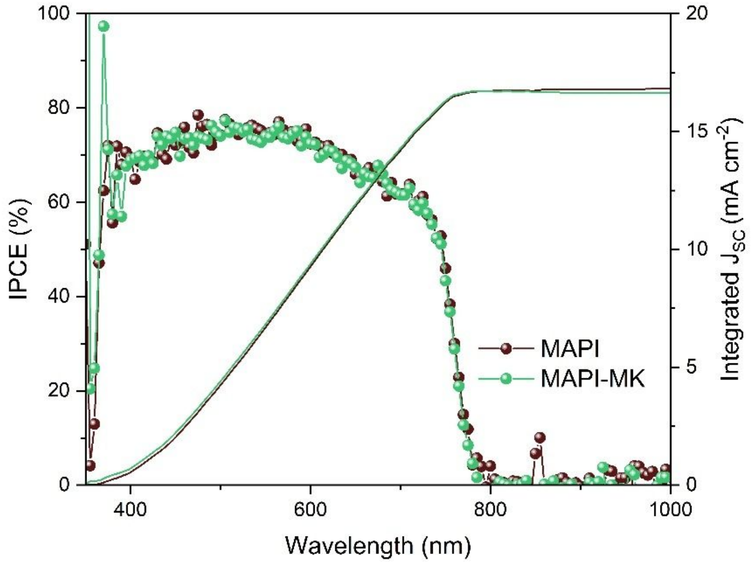

Figure S13. IPCE of manually fabricated mesoscopic solar cells with or without the MK245 dye in the precursor solution.

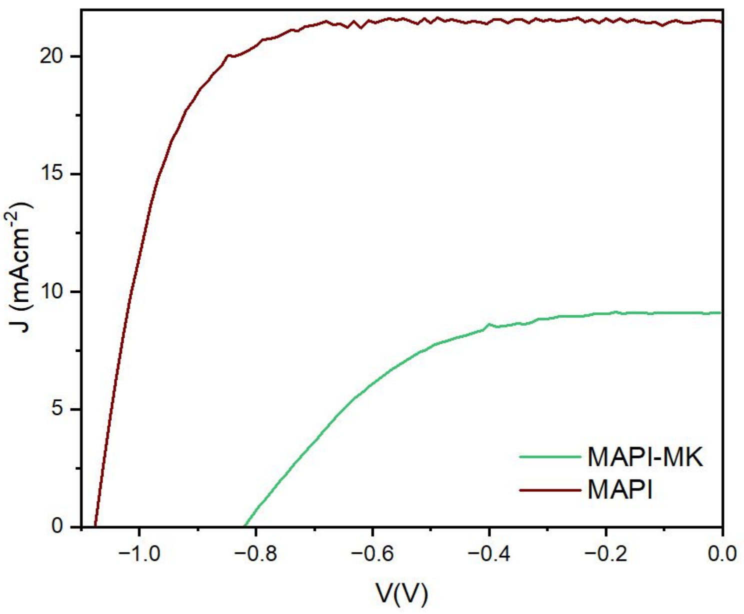

Figure S14. *J-V* curves of the manually fabricated solar cells with the configuration of FTO/c-TiO_2/mTiO_2/perovskite (with or without 3 mM MK245)/Spiro-OMeTAD/Au.

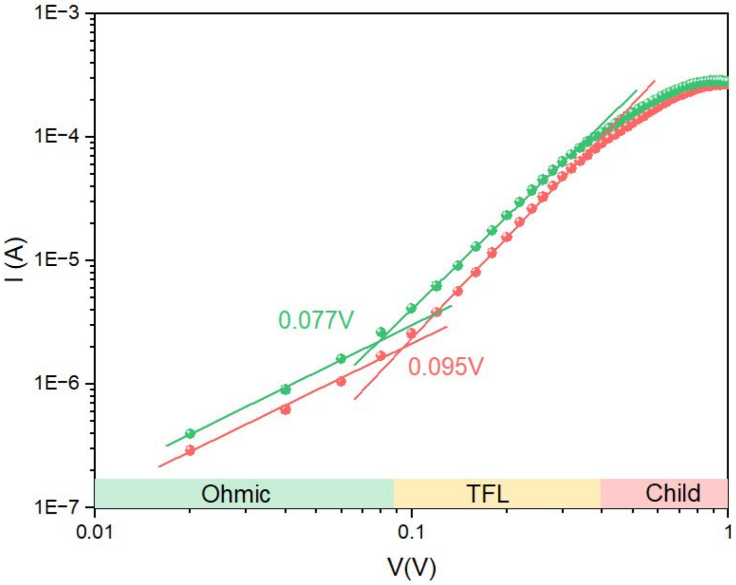

Figure S15. *I-V* curves of hole-only devices with the configuration ITO/PEDOT:PSS/perovskite (with or without MK245)/Spiro-OMeTAD/Au. Red: intrinsic perovskite; Green: with 3 mM MK245 dye in the precursor solution.

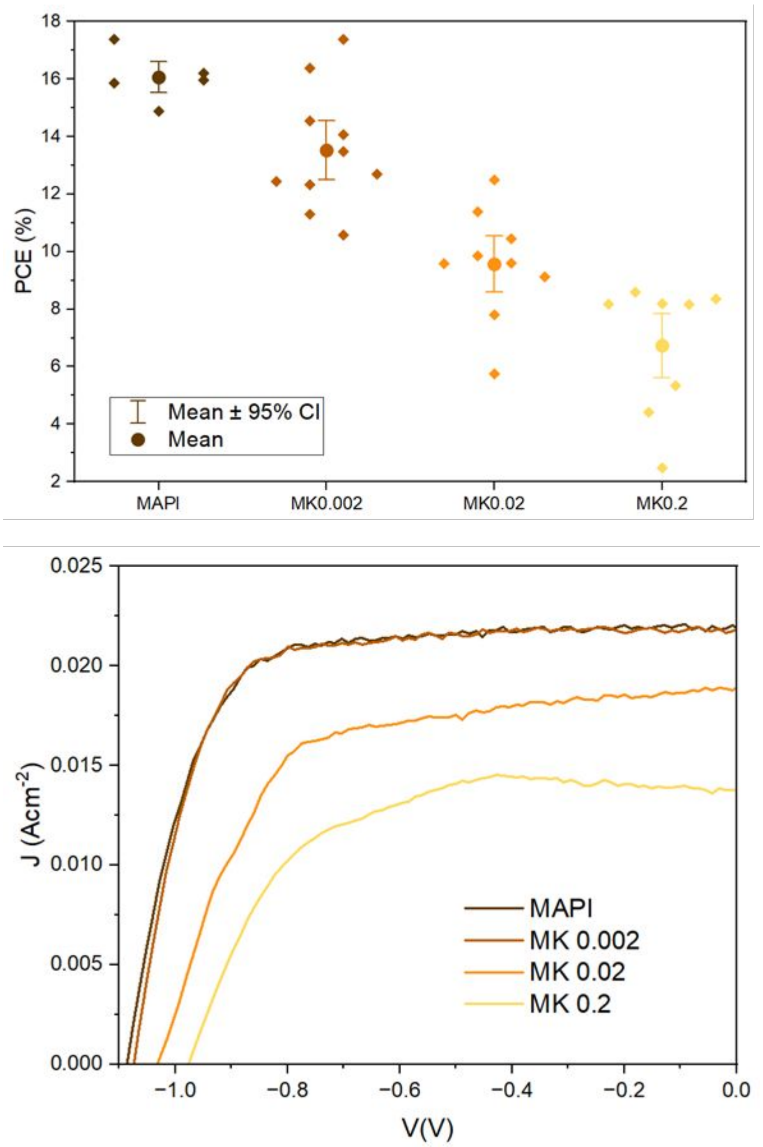

Figure S16. Top: PCE of n-i-p PSCs with intrinsic MAPI or MAPI coated by the MK245 dye of different concentrations (mg/mL). Bottom: *J-V* curves (reverse scan) of the champion device for each condition.

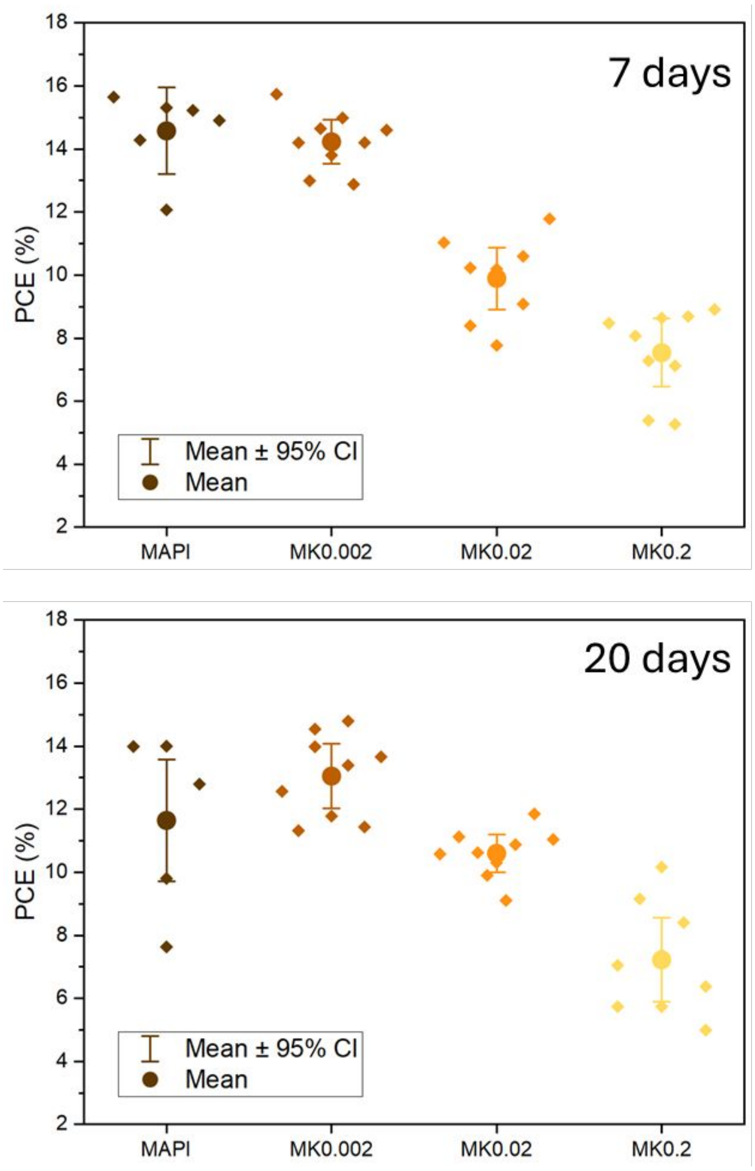

Figure S17. PCE of n-i-p PSCs with intrinsic MAPI or MAPI coated by the MK245 dye of different concentrations (mg/mL), investigated after 7 days and 20 days storage, respectively, under ambient condition (20 °C, 40-60% RH).

Supplementary Data

Table S9 Photovoltaic performance of solar cells on substrate 1, obtained from forward scans in the robotic solar-cell fabrication-evaluation workflow.

Label of cell	Cycle 1 scan 1	Cycle 1 scan 2	Cycle 2 scan 1	Cycle 2 scan 2	Cycle 3 scan 1	Cycle 3 scan 2
			**PCE (%)**			
0-1	7.38	8.46	/	/	/	/
0-2	7.38	8.23	9.33	10.47	10.49	11.81
1-1	8.84	9.25	9.91	10.90	10.65	11.77
1-2	8.11	8.89	9.54	10.61	10.49	11.59
2-1	6.22	6.85	8.85	9.74	10.04	11.01
2-2	9.79	10.12	11.99	12.98	13.02	14.19
3-1	9.68	10.52	12.72	13.50	13.99	14.85
3-2	9.37	9.74	12.40	13.26	13.54	14.30
4-1	7.65	8.18	9.29	9.99	10.34	11.04
4-2	7.77	8.44	9.87	10.51	10.91	11.61
5-1	9.44	10.03	11.96	12.63	13.15	13.83
5-2	10.03	10.58	11.84	12.60	12.93	13.66
6-1	2.96	3.11	3.67	3.81	4.07	4.25
6-2	1.28	1.85	2.68	2.85	3.48	3.72
7-1	3.09	4.32	4.57	4.76	4.87	5.10
7-2	4.76	4.96	4.60	4.86	5.11	5.37
			**J_{SC} (mA cm^{-2})**			
0-1	0.09	0.10	/	/	/	/
0-2	0.10	0.10	0.11	0.12	0.11	0.12
1-1	0.11	0.11	0.11	0.12	0.11	0.12
1-2	0.10	0.10	0.11	0.11	0.11	0.12
2-1	0.09	0.09	0.11	0.11	0.10	0.11
2-2	0.11	0.11	0.12	0.13	0.12	0.13
3-1	0.11	0.11	0.13	0.13	0.13	0.13
3-2	0.10	0.10	0.13	0.13	0.13	0.13
4-1	0.10	0.10	0.11	0.11	0.11	0.11
4-2	0.10	0.10	0.12	0.12	0.12	0.12
5-1	0.10	0.10	0.12	0.13	0.13	0.13
5-2	0.12	0.12	0.13	0.13	0.13	0.13
6-1	0.04	0.04	0.05	0.05	0.06	0.06
6-2	0.02	0.03	0.05	0.05	0.06	0.06
7-1	0.05	0.06	0.06	0.06	0.07	0.07
7-2	0.07	0.07	0.07	0.07	0.07	0.08

			**V_{OC} (V)**			
0-1	0.51	0.52	/	/	/	/
0-2	0.51	0.52	0.53	0.54	0.55	0.56
1-1	0.51	0.53	0.52	0.54	0.55	0.56
1-2	0.50	0.51	0.51	0.52	0.53	0.54
2-1	0.50	0.51	0.52	0.54	0.55	0.56
2-2	0.53	0.54	0.54	0.56	0.56	0.57
3-1	0.54	0.55	0.55	0.57	0.57	0.58
3-2	0.54	0.55	0.57	0.58	0.59	0.60
4-1	0.50	0.51	0.50	0.51	0.52	0.53
4-2	0.50	0.52	0.50	0.52	0.52	0.54
5-1	0.54	0.55	0.55	0.57	0.57	0.58
5-2	0.52	0.53	0.53	0.54	0.55	0.56
6-1	0.46	0.48	0.49	0.51	0.51	0.52
6-2	0.44	0.47	0.47	0.48	0.49	0.50
7-1	0.48	0.49	0.49	0.51	0.51	0.52
7-2	0.47	0.50	0.49	0.51	0.50	0.51
			**FF**			
0-1	0.39	0.42	/	/	/	/
0-2	0.38	0.41	0.40	0.41	0.45	0.45
1-1	0.40	0.42	0.42	0.43	0.44	0.44
1-2	0.41	0.44	0.43	0.45	0.46	0.46
2-1	0.37	0.39	0.40	0.42	0.44	0.46
2-2	0.41	0.43	0.45	0.46	0.48	0.49
3-1	0.40	0.43	0.45	0.45	0.47	0.48
3-2	0.43	0.45	0.43	0.45	0.45	0.46
4-1	0.39	0.41	0.42	0.44	0.45	0.46
4-2	0.39	0.41	0.43	0.44	0.45	0.46
5-1	0.43	0.45	0.44	0.45	0.46	0.47
5-2	0.42	0.44	0.43	0.45	0.46	0.47
6-1	0.37	0.37	0.38	0.37	0.36	0.36
6-2	0.31	0.34	0.29	0.29	0.30	0.30
7-1	0.33	0.39	0.38	0.37	0.36	0.36
7-2	0.38	0.37	0.35	0.34	0.35	0.35

Table S10 Photovoltaic performance of solar cells on substrate 2, obtained from forward scans in the robotic solar-cell fabrication-evaluation workflow.

Label of cell	Cycle 1 scan 1	Cycle 1 scan 2	Cycle 2 scan 1	Cycle 2 scan 2	Cycle 3 scan 1	Cycle 3 scan 2
			**PCE (%)**			
7-1	3.39	3.95	5.78	5.92	5.41	5.81
7-2	4.35	5.54	6.25	6.58	6.05	6.54
6-1	7.96	8.78	8.47	9.16	8.93	9.69
6-2	4.52	5.01	5.65	6.11	6.55	7.08
5-1	10.59	11.23	11.48	12.06	11.74	12.23
5-2	8.53	9.06	11.20	11.87	11.93	12.63
4-1	6.91	7.57	9.80	10.11	10.14	10.52
4-2	9.12	9.87	9.83	10.58	10.32	10.79
3-1	12.65	13.34	13.16	13.60	12.68	13.42
3-2	12.14	12.91	12.73	13.33	13.76	14.29
2-1	10.16	11.05	11.76	12.27	12.46	13.01
2-2	8.47	8.93	9.87	10.34	10.60	11.08
1-1	9.32	9.95	10.05	10.60	10.92	11.43
1-2	9.58	9.90	9.04	9.39	11.91	12.34
0-1	7.77	7.86	0.72	1.17	0.39	9.01
0-2	7.10	7.50	8.31	8.05	9.22	9.59
			**J_{SC} (mA cm^{-2})**			
7-1	0.04	0.05	0.06	0.06	0.06	0.07
7-2	0.06	0.06	0.07	0.07	0.07	0.07
6-1	0.08	0.08	0.08	0.09	0.09	0.09
6-2	0.06	0.06	0.08	0.08	0.08	0.08
5-1	0.09	0.09	0.10	0.10	0.10	0.10
5-2	0.08	0.08	0.10	0.10	0.10	0.10
4-1	0.07	0.07	0.09	0.09	0.09	0.09
4-2	0.08	0.08	0.09	0.09	0.09	0.09
3-1	0.11	0.11	0.12	0.12	0.12	0.12
3-2	0.11	0.11	0.12	0.12	0.12	0.12
2-1	0.10	0.10	0.11	0.11	0.11	0.11
2-2	0.08	0.08	0.10	0.10	0.10	0.11
1-1	0.09	0.09	0.10	0.10	0.10	0.10
1-2	0.09	0.09	0.10	0.10	0.10	0.10
0-1	0.08	0.08	0.02	0.03	0.01	0.10
0-2	0.07	0.07	0.09	0.09	0.09	0.09
			**V_{OC} (V)**			
7-1	0.44	0.48	0.49	0.51	0.52	0.53

7-2	0.47	0.50	0.49	0.51	0.53	0.54
6-1	0.49	0.51	0.49	0.51	0.52	0.53
6-2	0.48	0.50	0.50	0.52	0.53	0.54
5-1	0.53	0.55	0.53	0.55	0.55	0.56
5-2	0.51	0.53	0.52	0.55	0.55	0.56
4-1	0.49	0.51	0.49	0.52	0.53	0.55
4-2	0.49	0.52	0.50	0.52	0.53	0.55
3-1	0.52	0.54	0.52	0.54	0.55	0.56
3-2	0.52	0.53	0.52	0.54	0.54	0.55
2-1	0.51	0.53	0.51	0.53	0.54	0.55
2-2	0.48	0.50	0.49	0.51	0.51	0.53
1-1	0.48	0.51	0.50	0.52	0.53	0.55
1-2	0.51	0.52	0.53	0.55	0.56	0.57
0-1	0.48	0.50	0.53	0.54	0.54	0.54
0-2	0.47	0.49	0.49	0.51	0.52	0.53
			**FF**			
7-1	0.43	0.44	0.49	0.47	0.45	0.42
7-2	0.42	0.44	0.48	0.48	0.44	0.43
6-1	0.52	0.57	0.52	0.53	0.51	0.52
6-2	0.40	0.42	0.38	0.39	0.38	0.40
5-1	0.56	0.57	0.57	0.58	0.56	0.56
5-2	0.55	0.57	0.55	0.56	0.54	0.55
4-1	0.55	0.57	0.57	0.56	0.52	0.52
4-2	0.55	0.57	0.55	0.57	0.53	0.53
3-1	0.56	0.58	0.54	0.54	0.48	0.50
3-2	0.54	0.57	0.52	0.52	0.53	0.53
2-1	0.52	0.53	0.52	0.52	0.51	0.52
2-2	0.55	0.56	0.51	0.52	0.51	0.50
1-1	0.54	0.56	0.50	0.52	0.50	0.51
1-2	0.52	0.54	0.42	0.42	0.51	0.53
0-1	0.51	0.51	0.18	0.20	0.17	0.43
0-2	0.53	0.54	0.49	0.45	0.48	0.50

Table S11 Photovoltaic performance of the manually fabricated mesoscopic solar cells. Averages were calculated from 4 devices for each condition.

Sample	PCE (%)	J_{SC} (mA cm^{-2})	V_{OC} (V)	FF
		Day 1		
MAPI	8.25	15.06	0.92	0.59
MAPI-MK	7.87	13.40	0.91	0.64
		Day 7		
MAPI	7.91	13.84	0.90	0.64
MAPI-MK	7.86	14.64	0.88	0.61
		Day 20		
MAPI	5.53	13.07	0.81	0.53
MAPI-MK	7.77	14.00	0.82	0.68

Table S12 Photovoltaic performance of the manually fabricated n-i-p PSCs with intrinsic MAPI or MAPI coated by the MK245 dye of different concentrations (mg/mL). Averages were calculated from 6 devices with intrinsic MAPI and 9 devices for each condition of the MK245 dye treatment.

Sample	PCE (%)	J_{SC} (mA cm^{-2})	V_{OC} (V)	FF
		Day 1		
MAPI	16.06	21.24	1.08	0.70
MK0.002	13.77	20.84	1.06	0.62
MK0.02	9.57	15.84	1.01	0.60
MK0.2	6.73	11.88	0.97	0.57
		Day 7		
MAPI	14.58	20.65	1.08	0.65
MK0.002	14.23	20.36	1.07	0.66
MK0.02	9.90	16.57	1.02	0.59
MK0.2	7.54	12.98	0.99	0.58
		Day 20		
MAPI	11.65	20.44	1.07	0.53
MK0.002	13.05	20.88	1.06	0.59
MK0.02	10.60	17.76	1.02	0.59
MK0.2	7.22	14.07	1.00	0.51

Reference:

(1) Lei, B.; Svensson, P. H.; Yushmanov, P.; Kloo, L. AURORA - An Automatic Robotic Platform for Materials Discovery. *ACS Appl. Mater. Interfaces* **2025**, *17* (18), 26701–26709. https://doi.org/10.1021/acsami.5c02605.

(2) Frisch, M. J.; Trucks, G. W.; Schlegel, H. B.; Scuseria, G. E.; Robb, M. A.; Cheeseman, J. R.; Scalmani, G.; Barone, V.; Petersson, G. A.; Nakatsuji, H.; Li, X.; Caricato, M.; Marenich, A. V.; Bloino, J.; Janesko, B. G.; Gomperts, R.; Mennucci, B.; Hratchian, H. P.; Ortiz, J. V.; Izmaylov, A. F.; Sonnenberg, J. L.; Williams-Young, D.; Ding, F.; Lipparini, F.; Egidi, F.; Goings, J.; Peng, B.; Petrone, A.; Henderson, T.; Ranasinghe, D.; Zakrzewski, V. G.; Gao, J.; Rega, N.; Zheng, G.; Liang, W.; Hada, M.; Ehara, M.; Toyota, K.; Fukuda, R.; Hasegawa, J.; Ishida, M.; Nakajima, T.; Honda, Y.; Kitao, O.; Nakai, H.; Vreven, T.; Throssell, K.; Montgomery, J. A. Jr.; Peralta, J. E.; Ogliaro, F.; Bearpark, M. J.; Heyd, J. J.; Brothers, E. N.; Kudin, K. N.; Staroverov, V. N.; Keith, T. A.; Kobayashi, R.; Normand, J.; Raghavachari, K.; Rendell, A. P.; Burant, J. C.; Iyengar, S. S.; Tomasi, J.; Cossi, M.; Millam, J. M.; Klene, M.; Adamo, C.; Cammi, R.; Ochterski, J. W.; Martin, R. L.; Morokuma, K.; Farkas, O.; Foresman, J. B.; Fox, D. J. Gaussian 16 Revision C.01, 2016.

(3) Becke, A. D. Density-functional Thermochemistry. III. The Role of Exact Exchange. *The Journal of Chemical Physics* **1993**, *98* (7), 5648–5652. https://doi.org/10.1063/1.464913.

(4) Yanai, T.; Tew, D. P.; Handy, N. C. A New Hybrid Exchange–Correlation Functional Using the Coulomb-Attenuating Method (CAM-B3LYP). *Chemical Physics Letters* **2004**, *393* (1), 51–57. https://doi.org/10.1016/j.cplett.2004.06.011.

(5) Perdew, J. P.; Wang, Y. Accurate and Simple Analytic Representation of the Electron-Gas Correlation Energy. *Phys. Rev. B* **1992**, *45* (23), 13244–13249. https://doi.org/10.1103/PhysRevB.45.13244.

(6) Chai, J.-D.; Head-Gordon, M. Long-Range Corrected Hybrid Density Functionals with Damped Atom–Atom Dispersion Corrections. *Physical Chemistry Chemical Physics* **2008**, *10* (44), 6615–6620. https://doi.org/10.1039/B810189B.

(7) Zhao, Y.; Truhlar, D. G. The M06 Suite of Density Functionals for Main Group Thermochemistry, Thermochemical Kinetics, Noncovalent Interactions, Excited States, and Transition Elements: Two New Functionals and Systematic Testing of Four M06-Class Functionals and 12 Other Functionals. *Theor Chem Account* **2008**, *120* (1), 215–241. https://doi.org/10.1007/s00214-007-0310-x.

(8) COHEN, A. J.; HANDY, N. C. Dynamic Correlation. *Molecular Physics* **2001**, *99* (7), 607–615. https://doi.org/10.1080/00268970010023435.

(9) Tomasi, J.; Mennucci, B.; Cammi, R. Quantum Mechanical Continuum Solvation Models. *Chem. Rev.* **2005**, *105* (8), 2999–3094. https://doi.org/10.1021/cr9904009.

(10) Peterson, K. A.; Shepler, B. C.; Figgen, D.; Stoll, H. On the Spectroscopic and Thermochemical Properties of ClO, BrO, IO, and Their Anions. *J. Phys. Chem. A* **2006**, *110* (51), 13877–13883. https://doi.org/10.1021/jp065887l.

(11) Metz, B.; Stoll, H.; Dolg, M. Small-Core Multiconfiguration-Dirac–Hartree–Fock-Adjusted Pseudopotentials for Post-d Main Group Elements: Application to PbH and PbO. *The Journal of Chemical Physics* **2000**, *113* (7), 2563–2569. https://doi.org/10.1063/1.1305880.

(12) Peterson, K. A. Systematically Convergent Basis Sets with Relativistic Pseudopotentials. I. Correlation Consistent Basis Sets for the Post-d Group 13–15 Elements. *The Journal of Chemical Physics* **2003**, *119* (21), 11099–11112. https://doi.org/10.1063/1.1622923.
